# Predation upon Hatchling Dinosaurs by a New Snake from the Late Cretaceous of India

**DOI:** 10.1371/journal.pbio.1000322

**Published:** 2010-03-02

**Authors:** Jeffrey A. Wilson, Dhananjay M. Mohabey, Shanan E. Peters, Jason J. Head

**Affiliations:** 1 Museum of Paleontology and Department of Geological Sciences, University of Michigan, Ann Arbor, Michigan, United States of America; 2 Geological Survey of India (Central Region), Palaeontology Division, Nagpur, India; 3 Department of Geology and Geophysics, University of Wisconsin-Madison, Madison, Wisconsin, United States of America; 4 Department of Biology, University of Toronto, Mississauga, Mississauga, Ontario, Canada; University of Bristol, United Kingdom

## Abstract

Derived large-mouthed snakes (macrostomatans) possess numerous specializations in their skull and lower jaws that allow them to consume large vertebrate prey. In contrast, basal snakes lack these adaptations and feed primarily on small prey items. The sequence of osteological and behavioral modifications involved in the evolution of the macrostomatan condition has remained an open question because of disagreement about the origin and interrelationships of snakes, the paucity of well-preserved early snake fossils on many continental landmasses, and the lack of information about the feeding ecology of early snakes. We report on a partial skeleton of a new 3.5-m-long snake, *Sanajeh indicus* gen. et sp. nov., recovered from Upper Cretaceous rocks of western India. *S. indicus* was fossilized in association with a sauropod dinosaur egg clutch, coiled around an egg and adjacent to the remains of a ca. 0.5-m-long hatchling. Multiple snake-egg associations at the site strongly suggest that *S. indicus* frequented nesting grounds and preyed on hatchling sauropods. We interpret this pattern as “ethofossil” preservation of feeding behavior. *S. indicus* lacks specializations of modern egg-eaters and of macrostomatans, and skull and vertebral synapomorphies place it in an intermediate position in snake phylogeny. *Sanajeh* and its large-bodied madtsoiid sister taxa *Yurlunggur camfieldensis* and *Wonambi naracoortensis* from the Neogene of Australia show specializations for intraoral prey transport but lack the adaptations for wide gape that characterize living macrostomatan snakes. The Dholi Dungri fossils are the second definitive association between sauropod eggs and embryonic or hatchling remains. New fossils from western India provide direct evidence of feeding ecology in a Mesozoic snake and demonstrate predation risks for hatchling sauropod dinosaurs. Our results suggest that large body size and jaw mobility afforded some non-macrostomatan snakes a greater diversity of prey items than previously suspected on the basis of extant basal snakes.

## Abstract 2

A new snake from Upper Cretaceous rocks in India is found with hatchling sauropod dinosaurs, demonstrating that large, gape-limited snakes were probably capable of taking in moderate-sized vertebrate prey.

## Author Summary

Snakes first appear in the fossil record towards the end of the dinosaur era, approximately 98 million years ago. Snake fossils from that time are fragmentary, usually consisting of parts of the backbone. Relatively complete snake fossils preserving skulls and occasionally hindlimbs are quite rare and have only been found in marine sediments in Afro-Arabia and Europe or in terrestrial sediments in South America. Early snake phylogeny remains controversial, in part because of the paucity of early fossils. We describe a new 3.5-m-long snake from the Late Cretaceous of western India that is preserved in an extraordinary setting—within a sauropod dinosaur nest, coiled around an egg and adjacent the remains of a ca. 0.5-m-long hatchling. Other snake-egg associations at the same site suggest that the new snake frequented nesting grounds and preyed on hatchling sauropods. We named this new snake *Sanajeh indicus* because of its provenance and its somewhat limited oral gape. *Sanajeh* broadens the geographical distribution of early snakes and helps resolve their phylogenetic affinities. We conclude that large body size and jaw mobility afforded some early snakes a greater diversity of prey items than previously suspected.

## Introduction

Snakes are limbless reptiles that first appeared in the fossil record in the middle of the Cretaceous, approximately 98 million years ago [1]. Most species of living snakes are macrostomatans, which consume large prey items using a specialized gape achieved via a posteriorly displaced jaw joint, increased cranial kinesis, and an elongated skull and lower jaws. The evolution of large-gape feeding in macrostomatans has remained controversial owing to the scarcity of Cretaceous snake specimens preserving cranial and postcranial remains. Phylogenetic interpretation of these early snake fossils as either basal to all living snakes or to its subgroup Macrostomata has polarized views on snake origins, interrelationships, and ancestral habitat [2]–[6].

**Figure 1:**
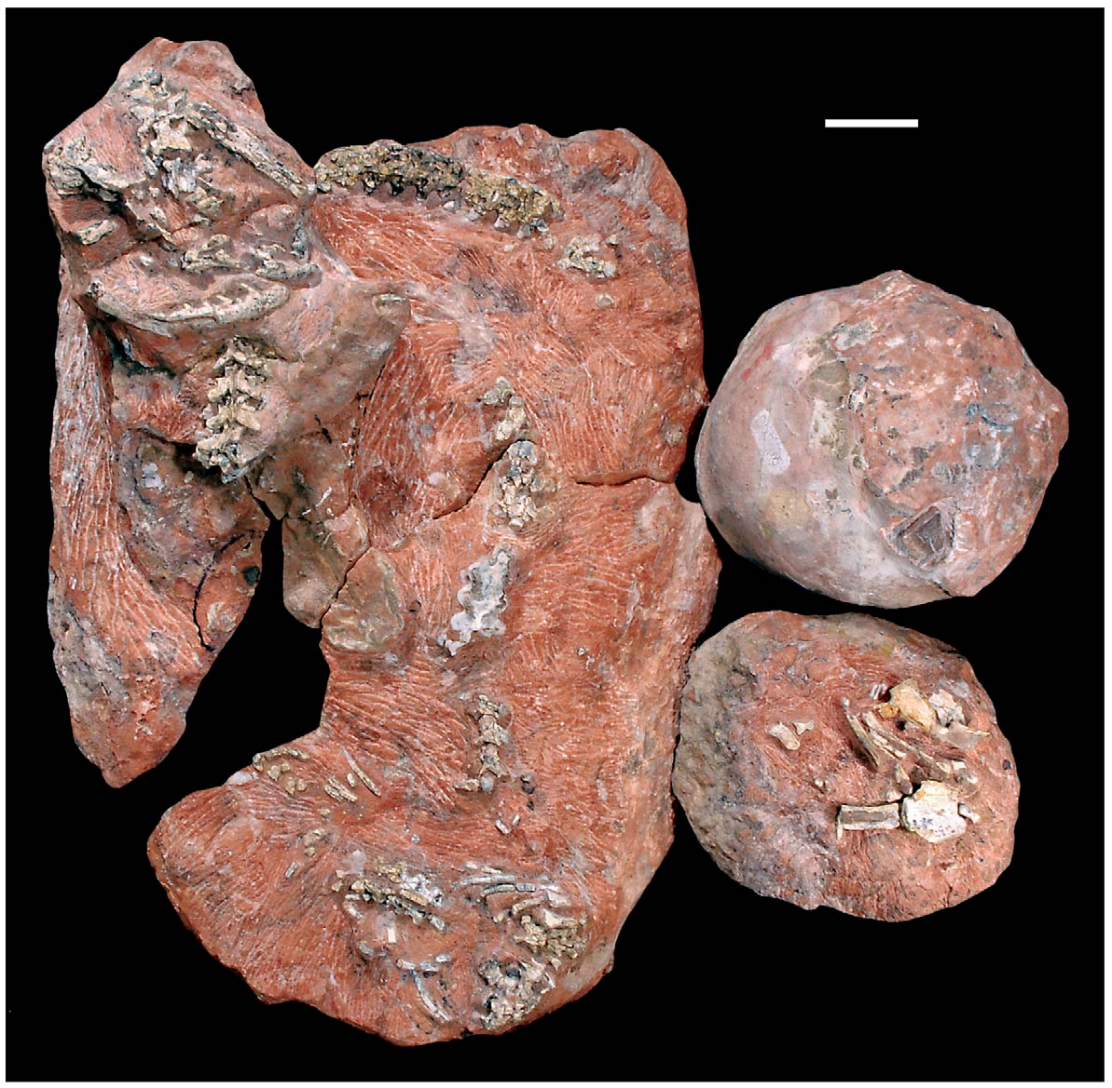
Fossil snake preserved within a sauropod dinosaur nesting ground. Photograph of blocks collected at Dholi Dungri, India preserving the snake *Sanajeh indicus*, n. gen. n. sp., in association with a partial clutch of three titanosaur eggs (oogenus *Megaloolithus*) and a titanosaur hatchling (GSI/GC/2901–2906). Beginning from the center of the lower portion of the photograph, the articulated skeleton of *Sanajeh* is coiled in a clockwise fashion around a crushed *Megaloolithus* egg (egg 3, at the junction of three blocks), with its skull resting on the topmost loop of the coil. The uncrushed *Megaloolithus* egg (egg 1) at right pertains to the same clutch, which would have contained six to 12 eggs. A second uncrushed *Megaloolithus* egg (egg 2) from the same clutch is still at the site. At lower right are the front quarters of a titanosaur hatchling, including elements of the thorax, shoulder girdle, and forelimb preserved in anatomical articulation. The titanosaur hatchling was approximately 0.5 m long, or one-seventh the length of *Sanajeh* (3.5 m long). No other sauropod bones were found at the site. Please see Figure 2 for interpretive map of specimen. Scale bar equals 5 cm.

**Figure 2:**
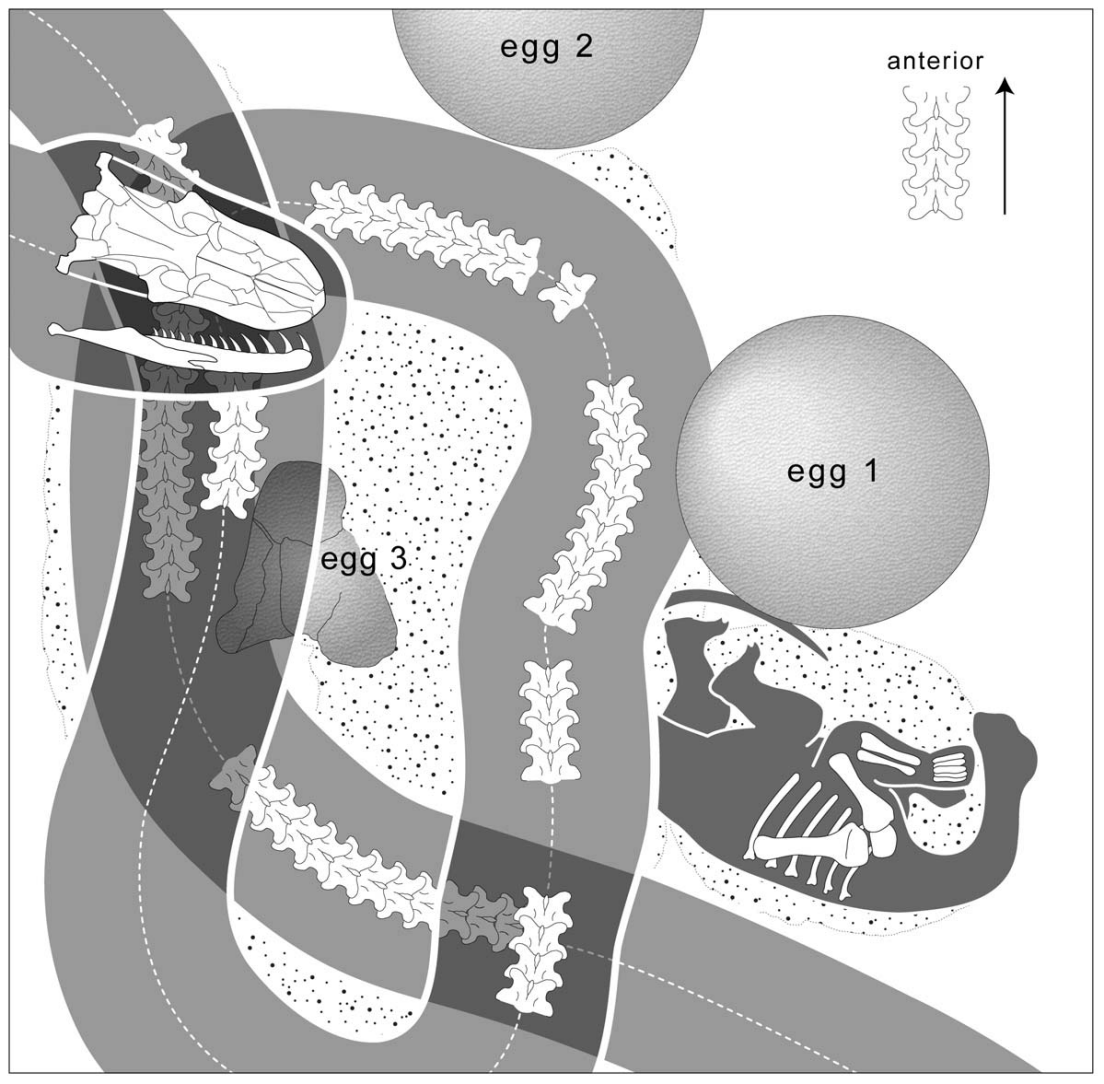
Fossil snake preserved within a sauropod dinosaur nesting ground. Interpretive map of blocks shown in Figure 1.

Here we describe an articulated snake fossil from uppermost Cretaceous horizons of Indo-Pakistan that is among the first such known from the subcontinent prior to the Miocene [7]. The new snake is preserved in an extraordinary setting—within a sauropod dinosaur nesting ground in association with eggs and a hatchling (Figures 1 and 2). The new fossils provide the first evidence, to our knowledge, of snake predation on hatchling dinosaurs and a rare example of non-dinosaurian predation on dinosaurs [8],[9]. Below we describe this new snake and its association with a sauropod egg clutch, resolve its phylogenetic relationships to other early snakes, and explore its implications for the evolution of wide-gape feeding in snakes and predation risks on sauropod dinosaurs.

## Results and Discussion

### Systematic Paleontology

Squamata Oppel 1811

Serpentes Linnaeus 1758

Alethinophidia Nopcsa 1923

Madtsoiidae Hoffstetter 1961

### *Sanajeh* gen. nov

urn:lsid:zoobank.org:act:AB09F42A-6E4E-4F96-8B32-60D4B9FA6FD6

#### Etymology

*Sanaj*, ancient, and *jeh*, gape (Sanskrit): named for the inferred feeding capabilities of this early snake.

#### Type species

*Sanajeh indicus* sp. nov.

#### Diagnosis

As for the species.

### *Sanajeh indicus* sp. nov

urn:lsid:zoobank.org:act:45E1476C-0BC1-4892-B4A9-B4D16530B43F

#### Etymology

From *sindu*, referring to the Indus River (Sanskrit): historically this river helped define the territory of the Indian subcontinent, whose name is derived from it.

#### Diagnosis

*S. indicus* is a ca. 3.5-m-long snake with a rectangular juxtastapedial recess, broad and squared-off supratemporal, wide and dorsally concave basioccipital posterolateral process, and precloacal vertebrae with small parazygantral foramina and thin, posterodorsally angled neural spines.

#### Holotype

GSI/GC/2901–2906, a nearly complete skull and lower jaws preserved in association with 72 precloacal vertebrae and ribs in five articulated sections (Figures 1 and S3). A cast of the specimen is housed at the University of Michigan Museum of Paleontology (UMMP 14265).

#### Locality, horizon, and age

*S. indicus* was collected from infratrappean calcareous sandstones of the Lameta Formation exposed near Dholi Dungri village in Gujarat, western India (23°08′ N; 73°23′ E; Figures S1, S5, and S6; see Texts S1 and S2). The Lameta Formation is considered Late Cretaceous (Maastrichtian) in age because of its close association with the overlying Deccan Traps, whose onset has been estimated to be 67.5 million years before present [10]. Lameta sediments were deposited in a variety of terrestrial environments from a semi-arid, tropical wet-dry climate [11] and preserve thousands of dinosaur eggs, hundreds of clutches, and scores of isolated bones [12],[13]. Eggs and bones are only found in association at the Dholi Dungri locality [14], where localized, episodic sediment transport events captured multiple associations of *S. indicus* with sauropod egg clutches. The sauropod eggs at Dholi Dungri were probably deposited in loose sediments in the proximity of a small drainage sourced from nearby Precambrian quartzite bedrock exposures, but much of the primary sedimentary structure and any evidence for a physically excavated nest were erased by extensive secondary soil-forming processes.

### Description of *Sanajeh indicus*

The skull and partial vertebral column of *Sanajeh* were found in articulation (Figures 1 and 2). On the basis of the length of the nearly complete skull (95 mm), we estimate total body length to be 3.5 m (Figure S7; Text S3).

**Figure 3:**
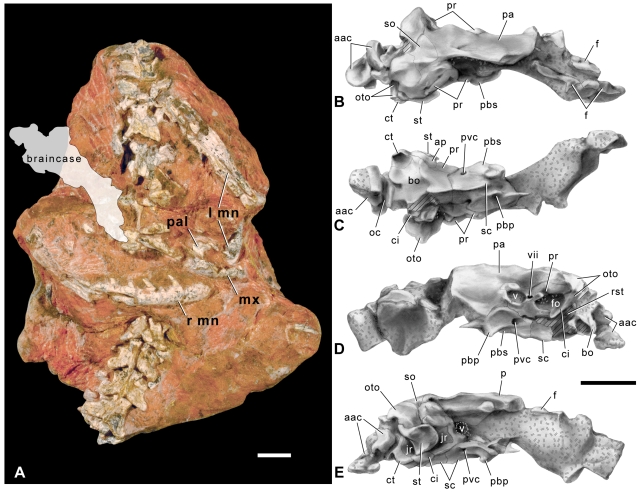
Skull of *S. indicus*, n. gen. n. sp. (A) Photograph of block GSI/GC/2903 showing the position of preserved cranial elements, which rest in near anatomical articulation upon a chain of vertebrae (anterior towards top). The braincase was removed from the block prior to final preparation, but its original position (gray tone) can be seen in Figure 1. (B–E) Half-tone drawings of the braincase in (B) dorsal; (C) ventral; (D) left lateral; and (E) right lateral views. aac, Atlas-axis complex; ap, accessory process of the crista interfenestralis; ci, crista interfenestralis; ct, crista tuberalis; f, frontal; fo, fenestra ovalis; jr, juxtastapedial recess; l, left; mn, mandible; mx, maxilla; oc, occipital condyle; oto, otooccipital; pa, parietal; pal, palatine; pbp, parabasisphenoid processes; pbs, parabasisphenoid; pr, prootic; pvc, posterior vidian canal; r, right; rst, recessus scalae tympani; sc, sagittal crest; so, supraoccipital; st, supratemporal; v–vii, openings for cranial nerves. Scale bars equal 2 cm.

Most of the jaws, palate, and braincase are preserved (Figures 3 and S8). The braincase is elongate, and its lateral surface bears two prominent openings that are separated by the I-shaped prootic (Figure 3D and 3E). These two openings, the trigeminal foramen and the juxtastapedial recess, house the cranial nerves associated with the jaws and the ear, respectively. The trigeminal foramen is the more anteriorly positioned of the two openings. It is bordered almost completely by the prootic but receives a small contribution to its anterior margin from the parietal. Like scolecophidians, *Dinilysia*, *Najash* [4], and the Australian madtsoiids *Wonambi* [15] and *Yurlunggur* [16], the trigeminal foramen is undivided. In alethinophidians, a laterosphenoid ossification subdivides the trigeminal foramen, separating maxillary and mandibular branches of cranial nerve V [17]. The more posterior, larger opening in the lateral wall of the braincase is the juxtastapedial recess, which is formed by the prootic and otooccipital. The juxtastapedial recess is subdivided into the fenestra ovalis, which houses the footplate of the stapes, and the recessus scalae tympani. The narrow crista interfenestralis separates these two openings and extends as an accessory process onto the ventral aspect of the skull (Figure 3C). Posteriorly, the juxtastapedial recess is bordered by a thick crista tuberalis, which begins on the otooccipital and extends posteroventrally to form the posterolateral corner of the ventral braincase. The architecture of the neurovascular openings within the recessus scalae tympani could not be examined, because this region is broken away on the left side of the skull and obscured by the supratemporal on the right. The short, broad supratemporal would have overlain the dorsal surface of the skull roof in articulation, as it does in macrostomatan snakes (Figure 3E). As in basal alethinophidian snakes such as *Xenopeltis*, the supratemporal has a wide articular surface for the quadrate on its lateral margin and a very short, free-ending posterior margin that does not extend posteriorly beyond the otic capsule. Importantly, the position of the quadrate articular facet, which is on the lateral surface of the supratemporal and located dorsal to the juxtastapedial recess, suggests that the jaw joint of *Sanajeh* was positioned lateral to the posterior margin of the braincase, as it is in basal snakes.

A prominent sagittal crest formed by the basioccipital and parabasisphenoid is present on the ventral aspect of the braincase (Figure 3C). This crest served as the insertion surface for muscles that moved the toothed bones of the palate (*m. protractor pterygoidei*) [18]. Paired parabasisphenoid processes project ventrally from the anterior end of this crest, as in *Wonambi*, boines, and pythonids. Arcuate crests extend posterolaterally from the posterior end of the crest, as in *Yurlunggur*, *Wonambi*, and some macrostomatans. A conspicuous opening for the posterior opening of the Vidian canal is preserved on the parabasisphenoid, but its anterior opening was not preserved. An enclosed Vidian canal is unique to squamates and carries the internal carotid artery and a branch of cranial nerve VII [17].

The facial and palatal portions of the skull are not as well preserved as the braincase and skull roof, but they are complete enough to estimate total skull length to be 95 mm. The maxilla is nearly complete and has a relatively short narial region. Its short, recurved anterior process and prominent dorsal process resemble those of anilioids. The dentary bears a single mental foramen, located near its anterior end, and a long posterior dentigerous process. Dentary teeth are broad and only slightly recurved, a condition more similar to anilioids than macrostomatans (Figure 3A).

**Figure 4:**
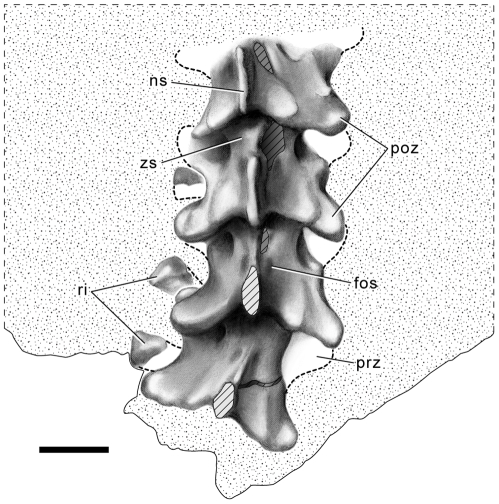
Precloacal vertebrae of *S. indicus*, n. gen. n. sp. Half-tone drawing of the four articulated vertebrae at the base of the block GSI/GC/2903. fos, fossa; ns, neural spine; poz, postzygapophysis; prz, prezygapophysis; ri, rib; zs, zygosphene. Scale bar equals 2 cm.

The vertebral column of *Sanajeh* is represented by precloacal vertebrae (Figures 4 and S9; Text S4). The wedge-and-notch zygosphene-zygantrum articulations are well developed, and the zygantrum is flanked by small parazygantral foramina on the posterior surface of the neural arch, as in *Najash* [4] and taxa referred to Madtsoiidae. The neural spines of *Sanajeh* are thin and strongly posteriorly angled, overhanging the shallow embayment between the postzygapophyses. Shallow fossae are present on either side of the neural spine. The prezygapophyses lack accessory processes, and the rib articulations (synapophyses) extend laterally beyond the margins of the prezygapophyses, both of which are characters present in madtsoiids [16].

### Snake–Dinosaur Association

The skeleton of *Sanajeh* was preserved in close association with three sauropod eggs of the oospecies *Megaloolithus dhoridungriensis* [19] and a partial sauropod hatchling (Figures 1, 2, and S10; Text S5). The eggs represent part of a single clutch, which typically contains six to 12 eggs at Dholi Dungri. No nest structure is preserved at Dholi Dungri nor any other Indian egg locality, owing to extensive postburial pedogenic modification of the entombing sediments [19]. The high porosity of the eggs at Dholi Dungri suggests that they were incubated in a nest covered by a layer of either vegetation or loose sediment [20]. The skull of *Sanajeh* rests atop a coil of the vertebral column, which wraps around three sides of a crushed egg (Figures 1 and 2). The two other eggs are uncrushed and unhatched, and we infer that the crushed egg encircled by the snake was exited by the sauropod hatchling found adjacent to it.

**Figure 5:**
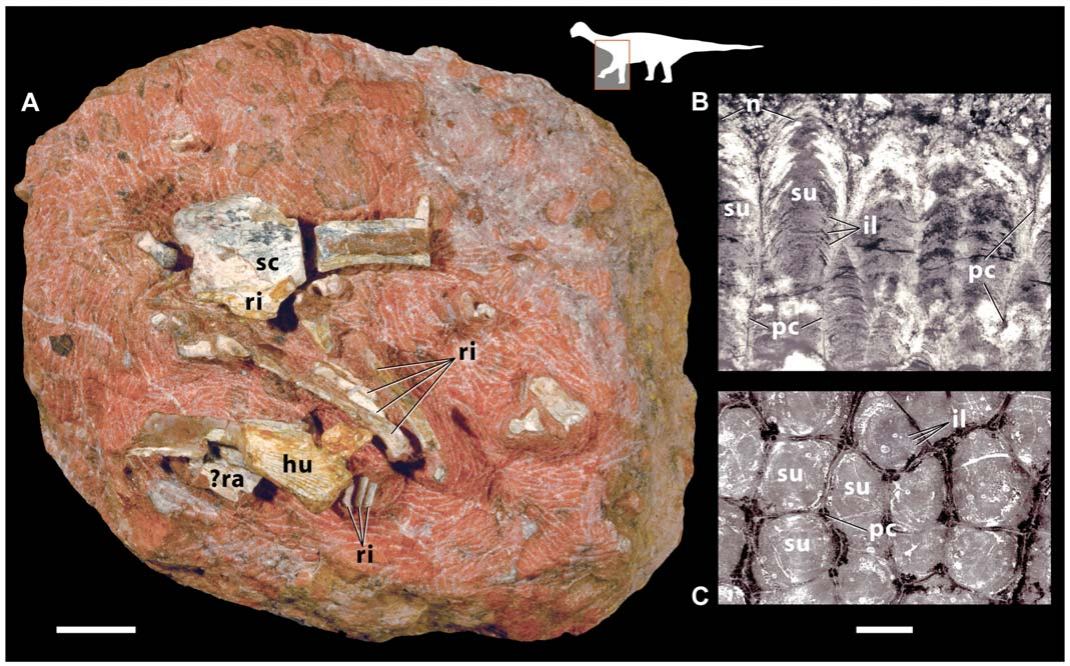
Titanosaur sauropod hatchling and egg. (A) Photograph of block GSI/GC/2904, showing elements of the anterior thorax and forelimb of the hatchling. The images at right are radial (B) and tangential (C) sections through an eggshell fragment removed from titanosaur egg 3 (from block GSI/GC/2905). External is towards the top in (B). hu, Humerus; il, incremental lines; n, node; pc, pore canal; ra, radius; ri, rib; sc, scapula; su, shell unit. Scale bar equals 2 cm for (A) and 500 µm for (B and C).

The sauropod hatchling is represented by a portion of the left side of the anterior thorax, a partial shoulder girdle, and a partial forelimb preserved in anatomical articulation (Figure 5). The hatchling bones are not completely ossified, but they can be confidently attributed to a sauropod dinosaur on the basis of the presence of a relatively large acromial region on the proximal scapula and a straight-shafted humerus [21]. The hatchling almost certainly is a titanosaur because no other sauropod lineage has been recovered from uppermost Cretaceous sediments in Indo-Pakistan or elsewhere [22]. The Dholi Dungri specimen is only the second definitive association between sauropod bones and eggs [23].

Multiple lines of evidence suggest that the snake-dinosaur association preserved at Dholi Dungri was the result of preservation of organisms “caught in the act” rather than a postmortem accumulation of independently transported elements. First, the pose of the snake with its skull resting atop a coil encircling a crushed egg is not likely to have resulted from the transport of two unassociated remains. Second, the high degree of articulation of the snake, hatchling, and crushed egg, as well as the excellent preservation of delicate cranial elements and intact, relatively undeformed eggs rule out substantial transport and are indicative of relatively rapid and deep burial. Third, our sedimentological analysis indicates that the site was located adjacent to a paleotopographic high that could have been the source of rapid sedimentation pulses as a result of storm-induced debris flows (see Text S2). Fourth, at least three individual snake specimens were found associated with sauropod eggs, suggesting active habitation of nests rather than postmortem transport.

The three associations of *Sanajeh* bones and *Megaloolithus* eggs found over a 25-m^2^ area, together with the sedimentological and taphonomic profile of the site, suggest “ethofossil” preservation—i.e., a record of typical behavior rather than of aberrant behavior or a fatal mistake [24]. We infer that *Sanajeh* actively frequented sauropod nesting environments and predated upon sauropod hatchlings. It is unlikely that *Sanajeh* consumed large, intact, rigid sauropod eggs (16 cm diameter, 2,145-cm^3^ volume), which greatly exceed its gape, because it lacks the cranial and vertebral adaptations for consumption of large eggs present in oophagous macrostomatans [25],[26]. However, it is possible that *Sanajeh* consumed contents of the sauropod eggs in a fashion resembling the non-macrostomatan snake *Loxocemus bicolor*, which is known to break eggs of the Olive Ridley sea turtle (*Lepidochelys olivacea*) by constriction and then ingest shell and contents with minimal loss [27]. In addition, *L. bicolor* is known to consume both eggs and hatchlings of the lizards *Ctenosaura* and *Iguana* [28],[29] and has a relatively flexible prey restraint repertoire [30]. Given the presence of theropod dinosaur eggs and smaller reptile eggs at the site (unpublished data), it is possible that a broad range of prey items supported a nest-plundering feeding strategy for *S. indicus*.

### Phylogenetic Relationships of *Sanajeh indicus*

**Figure 6:**
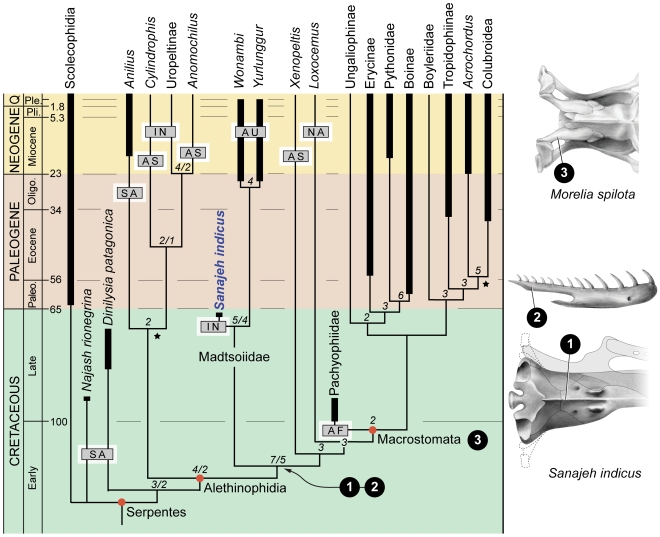
Calibrated phylogeny of snakes and evolution of wide-gape feeding. Adams consensus of the single most parsimonious trees derived from analyses employing Amphisbaenia and Varanoidea as outgroups. Topologies were identical except for the position of *Najash* relative to Scolecophidia and *Dinilysia*. Numbers at nodes indicate decay values greater than 1; where decay indices differ between analyses, both are reported (separated by a “/”). Trees rooted with Amphisbaenia have stronger support at basal nodes (see Text S6 for additional details). Half-tone drawings at right illustrate three innovations in the evolution of large gape in snakes. Basal alethinophidians such as *Sanajeh* acquired a prominent median ventral keel on the basioccipital and parabasisphenoid (1) and an elongate posterior dentary process (2), which suggest increased intraoral mobility. Macrostomatans evolved an elongate supratemporal bone (3) that increases gape by positioning the jaw joint well posterior of the occipital condyle. Geographic distributions (gray rectangles) indicate Gondwanan affinities for basal snakes, including an Indo-Australian distribution for the clade including *Sanajeh*, *Wonambi*, and *Yurlunggur*. Scolecophidia and Macrostomata possess a cosmopolitan distribution, and outgroup distributions are primarily Laurasian [15]. The taxonomic composition of Macrostomata follows [6] and [17]. Stars indicate first occurrences based on stem-group fossils [47],[48]. Abbreviations: AF, Africa; AS, Asia; AU, Australia; IN, India; NA, North America; SA, South America.

A phylogenetic analysis of 116 characters in 23 fossil and recent snake taxa resolves *S. indicus* as the sister taxon to the late Cenozoic Australian snakes *Wonambi* and *Yurlunggur* (Figure 6). The latter have been referred to as madtsoiids [15],[16], and we apply this name to the clade uniting *Sanajeh*, *Wonambi*, and *Yurlunggur* but note that additional phylogenetic investigation is needed to resolve whether this clade includes the giant, fragmentary South American, African, and Malagasy species that originally formed the basis for the group (e.g., *Madtsoia bai* and *M. madagascariensis*), or the numerous Cretaceous and Paleogene taxa that have subsequently been assigned to it based on vertebral morphology [31],[32]. Lengthy ghost lineages preceded the appearance of *Wonambi* and *Yurlunggur* in the fossil record, consistent with their hypothesized early origin on Gondwana [33]. Morphology of the braincase and mandibular suspensorium resolve the madtsoiids *Sanajeh*, *Yurlunggur*, and *Wonambi* as phylogenetically intermediate between narrow-gaped anilioids and wide-gaped macrostomatans. Our analysis does not support the proposition that the Australian madtsoiids *Wonambi* and *Yurlunggur* are closely related to the South American snakes *Dinilysia* and *Najash*, which are here resolved as basal snakes [34]. Although previous phylogenetic studies placed *Yurlunggur* and *Wonambi* as either basal snakes or derived macrostomatans [6],[16], the shortest trees for these alternative arrangements each require 21 additional evolutionary steps (Figure S12; see Text S6). We found only weak support for the monophyly of Anilioidea, which is not supported by molecular studies [35]. We found relatively strong support, in contrast, for a derived position for the limbed, marine pachyophiids, whose position is uncertain in other analyses [2]–[6].

### Evolution of Gape and Feeding in Early Snakes

Our phylogenetic analysis has important implications for the evolution of feeding in snakes (Figure 6). Basal snakes, which include scolecophidians and anilioids (uropeltines, *Anomochilus*, *Cylindrophis*, *Anilius*), possess a narrow oral gape and limited kinesis of the palatal bones. Their prey items are generally restricted to ant and termite larvae (scolecophidians) or annelids and small-bodied, often elongate limbless vertebrates such as amphisbaenians and caecilians (anilioids). This feeding ecology has been hypothesized to represent the plesiomorphic condition for snakes [36]. Conversely, derived macrostomatan snakes (boids, pythonids, caenophidians) evolved a specialized wide oral gape that allows them to consume a variety of relatively large-bodied prey items. Osteological specializations facilitating wide gape feeding in macrostomatans include posterior displacement of the jaw joint via an elongate, free-ending posterior process of the supratemporal bone, elongation of the lower jaws, and increased mobility of the tooth-bearing bones of the upper and lower jaws [36].

The evolutionary transition from narrow-gape feeding to wide-gape macrostomy has remained controversial owing to disagreement about the interrelationships of snakes and paucity of well-preserved fossils and ecological data for basal and early appearing snake taxa. *Sanajeh* possesses cranial characters that, combined with its depositional context and ecological associations, shed light on this transition (Figure 6). The short supratemporal and inferred broad, short quadrate indicate a narrow oral gape comparable to that of anilioids and *Xenopeltis*. However, the large insertion for *m. protractor pterygoidei* indicates powerful movement of the palatopterygoid bar during intraoral prey manipulation, as in derived macrostomatans [18]. The presence of a long posterior articular process of the dentary indicates extensive flexure of the intramandibular joint during intraoral prey transport, a condition *Wonambi*, *Yurlunggur*, and *Sanajeh* share with macrostomatans. Together, basicranial, mandibular, and suspensorial morphology indicate that expanded oral kinesis and complex intraoral mobility allowing for efficient intake of a variety of prey types and shapes preceded the evolution of a wide gape in snakes (Figure 6). Large body size combined with intraoral kinesis may have been a strategy that allowed gape-limited snakes such as *Sanajeh*, *Yurlunggur*, and *Wonambi* to consume large prey. On the basis of the feeding ecology of *Sanajeh* and the basal position of the large-bodied *Dinilysia*, we conclude that the high prey specificity and reduced cranial kinesis observed in extant basal snakes may not result from plesiomorphic gape-width restrictions, but may be specializations associated with fossoriality—especially miniaturization and habitat limitations on prey diversity [36].

### Predation Pressure on Hatchling Sauropods

Squamates (e.g., *Sphaerodactylus ariasae*, 0.014 kg) and sauropod dinosaurs (e.g., *Brachiosaurus brancai*, 38,000 kg) bracket reptile body mass range, which spans six orders of magnitude [37],[38]. The large body size (20–25 m) attained by the two titanosaur genera recognized from Indo-Pakistan, *Isisaurus* and *Jainosaurus* [39], may have been an effective deterrent to predators, but hatchlings were likely vulnerable to predation by organisms too small to prey upon adults. Large numbers of offspring [40] and accelerated growth rates [41],[42] may have offset losses of hatchlings to snake predation. “Ethofossil” preservation at Dholi Dungri captured an early instant in sauropod ontogeny when a 3.5-m-long snake maintained a body size advantage. Although at least one of the titanosaur species from India bore osteoderms, these elements probably did not form a shield of armor [43] and have never been recorded in hatchlings [44], which are poorly ossified. Because living derived macrostomatan snakes of comparable length ingest prey weighing much less than 10 kg [45], titanosaur hatchlings were probably free of risk of predation by *Sanajeh*-sized snakes before the end of their first year of growth.

## Methods

### History of the Discovery

The specimen described in this paper was discovered by one of us (DMM) in 1984 near the village of Dholi Dungri in western India. The specimen was collected using hand tools and removed as a series of blocks. The specimen was covered with a preservative but not subjected to chemical or mechanical preparation at the time. The initial descriptive paper [14], written well before the specimen was prepared, interpreted the specimen as a hatchling sauropod dinosaur preserved inside a nest (Figure S2). Although identification of sauropod egg and hatchling sauropod limb bones was correct, the vertebrae were incorrectly identified. S.L. Jain [12] was the first to correctly identify the vertebrae preserved on the main block as pertaining to a snake, an observation that went largely unnoticed and was never followed by detailed study. In 2001, one of us (JAW) reexamined the specimen and independently arrived at the same conclusion that Jain made 12 years earlier. Further study in the GSI collections by DMM and JAW uncovered a block that had been collected with the original specimen but was never described and, as a consequence, had been dissociated from it. That block has a snap-fit on the other blocks and preserves vertebrae that complete the snake's loop around the crushed egg (Figures S3 and S4; Text S1).

### Field Methods

Additional field reconnaissance conducted by the authors in 2007 relocated the original site at Dholi Dungri and collected additional geological and paleontological data. These included a detailed stratigraphic and sedimentological investigation of the site (Text S2) and the discovery of multiple associations between *Sanajeh* and sauropod eggs.

### Preparation

In 2004, the specimen was brought to the University of Michigan Museum of Paleontology, where it was prepared using a combination of chemical and mechanical techniques. The original lacquer preservative was removed from each block using Zip-Strip and then subjected to 3% formic acid for approximately 2–3 h, which weakened calcareous cement. Each block was then mechanically prepared using a micro-airscribe and needles to uncover the “up” surface of the bones. The blocks were fit together as they were found in the field and then molded and cast. The snake braincase and sauropod scapula and humerus were then fully freed from the matrix.

### Nomenclatural Acts

The electronic version of this document does not represent a published work according to the International Code of Zoological Nomenclature (ICZN), and hence the nomenclatural acts contained in the electronic version are not available under that Code from the electronic edition. Therefore, a separate edition of this document was produced by a method that assures numerous identical and durable copies, and those copies were simultaneously obtainable (from the publication date noted on the first page of this article) for the purpose of providing a public and permanent scientific record, in accordance with Article 8.1 of the Code. The separate print-only edition is available on request from PLoS by sending a request to PLoS Biology, 185 Berry Street, Suite 3100, San Francisco, CA 94107, USA along with a check for $10 (to cover printing and postage) payable to “Public Library of Science.”

The online version of the article is archived and available from the following digital repositories: PubMedCentral (www.pubmedcentral.nih.gov/), LOCKSS (http://www.lockss.org/lockss/), and Deep Blue at the University of Michigan (http://deepblue.lib.umich.edu/).

In addition, the genus and species names established herein have been registered in ZooBank, the proposed online registration system for the ICZN. The ZooBank LSIDs (Life Science Identifiers) can be resolved and the associated information viewed through any standard web browser by appending the LSID to the prefix “http://zoobank.org/”. The LSID for genus is: AB09F42A-6E4E-4F96-8B32-60D4B9FA6FD6 and the LSID for the species is:45E1476C-0BC1-4892-B4A9-B4D16530B43F.

### Body Size Estimation

We estimated body size of *S. indicus* by constructing a regression model of total body length onto skull length for crown-group snakes (Figure S7; see Text S3).

### Phylogenetic Analysis

Characters used in this analysis come from evaluation of the two most recent comprehensive morphological analyses of snake phylogeny [4],[16] and original specimen observations by JJH (see Text S6 for examined specimens). We used Amphisbaenia and Varanoidea as alternative outgroups to snakes on the basis of the most recent comprehensive analysis of squamate relationships [46]. We derived our phylogeny using a heuristic parsimony search in PAUP* 4.0 b using 10,000 random addition sequence replications. For additional information about the analysis, matrix, character list, and constraint trees, please see Text S6.

## Supporting Information

Figure S1 **Geological map of rocks cropping out near the Dholi Dungri site in Kheda District, Gujarat (western India).** Drafted by DMM.
(0.58 MB TIF)

Figure S2 **Snake-egg-hatchling blocks collected at Dholi Dungri, Gujarat State, India.** This is a reproduction of a plate from [6], showing the initial state prior to preparation. Compare to Figure S3. Gray boxes indicate field numbers assigned to blocks. Scale is in centimeters.
(3.43 MB TIF)

Figure S3 **Fully prepared snake-egg-hatchling blocks.** Note addition of the “Gandhinagar block” (GSI/GS/2906) and the different orientation and position of the cranial block (GSI/GC/2903). Scale is in centimeters.
(4.70 MB TIF)

Figure S4 **“Gandhinagar” block (GSI/GC/2906).** This block preserves fragments of the crushed *Megaloolithus* egg and a chain of *Sanajeh* vertebrae connecting the series on blocks GSI/GC/2901 and GSI/GC/2902. This image shows the underside of the block shown in Figure S3. Scale equals 5 cm.
(2.84 MB TIF)

Figure S5 **Stratigraphic column for Dholi Dungri.** Section base is at 23° 07.754′ N, 73° 22.544′ E; terminus is at 23° 07.818′ N, 73° 22.544′ E. All unit contacts, with the exception of the boulder lag and Precambrian basement, are gradational. Lateral variability not reflected in this transect. Drafted by SEP.
(0.38 MB TIF)

Figure S6 **Stratigraphic and petrologic examples of the Lameta Formation at Dholi Dungri.** (A) Overview of section near *Sanajeh* discovery site. Cobble lag at base of photo represents an ephemeral Maastrichtian drainage (see Figure S5). Resistant bed at top of slope is in silcrete interval near top of section. (B) Base of section, above Proterozoic basement. Carbonate- and silica-cemented, poorly sorted sand with angular quartzite clasts. (C-D) Fossil-bearing interval. (C) Carbonate- and silica-cemented, poorly sorted sand with subrounded quartzite and vein-quartz clasts. (D) cross-section showing bone fragment (top center of image). (E) Near top of section. Pedogenic fabric characteristic of nodular caliche interval (see Figure S5). (F) Silcrete interval. Discontinuous, resistant veins are composed of silica cements. The Lameta Formation at Dholi Dungri has been extensively diagenetically modified by silcrete and calcrete pedogenesis, but there is evidence for episodic sedimentation near a paleotopographic bedrock high. It is possible that this sedimentation resulted in the preservation of the snake-nest association. Rupee coins in (B, C, and F) are 2.5 cm in diameter.
(4.75 MB TIF)

Figure S7 **Body length estimate for** ***S. indicus*** **.** An estimated skull length of 95 mm indicates a total body length (TBL) of 3.46 m.
(0.19 MB TIF)

Figure S8 **Braincase and skull roof** ***S. indicus*** **.** Photographs in right lateral (A), left lateral (B), dorsal (C), and ventral (D) views. Scale equals 5 cm.
(2.76 MB TIF)

Figure S9 **Articulated vertebrae of** ***S. indicus*** **.** Photographs of vertebrae on block GSI/GC/2902 (A) and block GSI/GC/2903 (B) in dorsal view. Scale equals 2 cm for both images.
(4.44 MB TIF)

Figure S10 ***Megaloolithus*** **eggshell histology.** Thin-sections of uncrushed (A) and crushed (B) eggshell from blocks GSI/GC/2906 and GSI/GC/2905, respectively. Scale equals 1 mm.
(6.69 MB TIF)

Figure S11 **Consensus of the single most parsimonious trees derived from analyses employing Amphisbaenia and Varanoidea as outgroups.** Topologies were identical except for the position of *Najash* relative to Scolecophidia and *Dinilysia*. Numbers at nodes indicate decay values greater than 1; where decay indices differ between analyses, both are reported (separated by a “/”). Trees rooted with Amphisbaenia have stronger support at basal nodes. Tree statistics are shown at lower right; n, number of trees; TL, treelength; CI, consistency index; RI, retention index; RC, rescaled consistency index; A, Amphisbaenia; V, Varanoidea.
(0.23 MB TIF)

Figure S12 **Constraint trees.** Top, basal positions of *Wonambi*, *Yurlunggur*, *Dinilysia*, and Pachyophiidae were fixed at base of tree (but with no specified relationship to one another); bottom, a sister-taxon relationship between *Wonambi* and Boinae was fixed. Constrained taxa are indicated with arrows. Dashed lines in top cladogram indicate unresolved nodes in strict consensus of five trees rooted by Amphisbaenia. Tree statistics are shown in boxes at lower right; abbreviations as in Figure S11, except: d, parsimony debt under topological constraints.
(0.36 MB TIF)

Text S1 **History of the discovery.**
(0.03 MB DOC)

Text S2 **Geological setting.**
(0.03 MB DOC)

Text S3 **Body size estimate for** ***S. indicus*** **.**
(0.02 MB DOC)

Text S4 **Additional anatomical description.**
(0.06 MB DOC)

Text S5 **Ootaxonomic affinities of eggs at Dholi Dungri.**
(0.03 MB DOC)

Text S6 **Phylogenetic analysis.**
(0.15 MB DOC)
