# Supplementary figures and images for: Predation upon Hatchling Dinosaurs by a New Snake from the Late Cretaceous of India
Source: PLoS Biol. 2010 Mar 2;8(3):e1000322. doi: 10.1371/journal.pbio.1000322 (PMC2830453; doi:10.1371/journal.pbio.1000322)

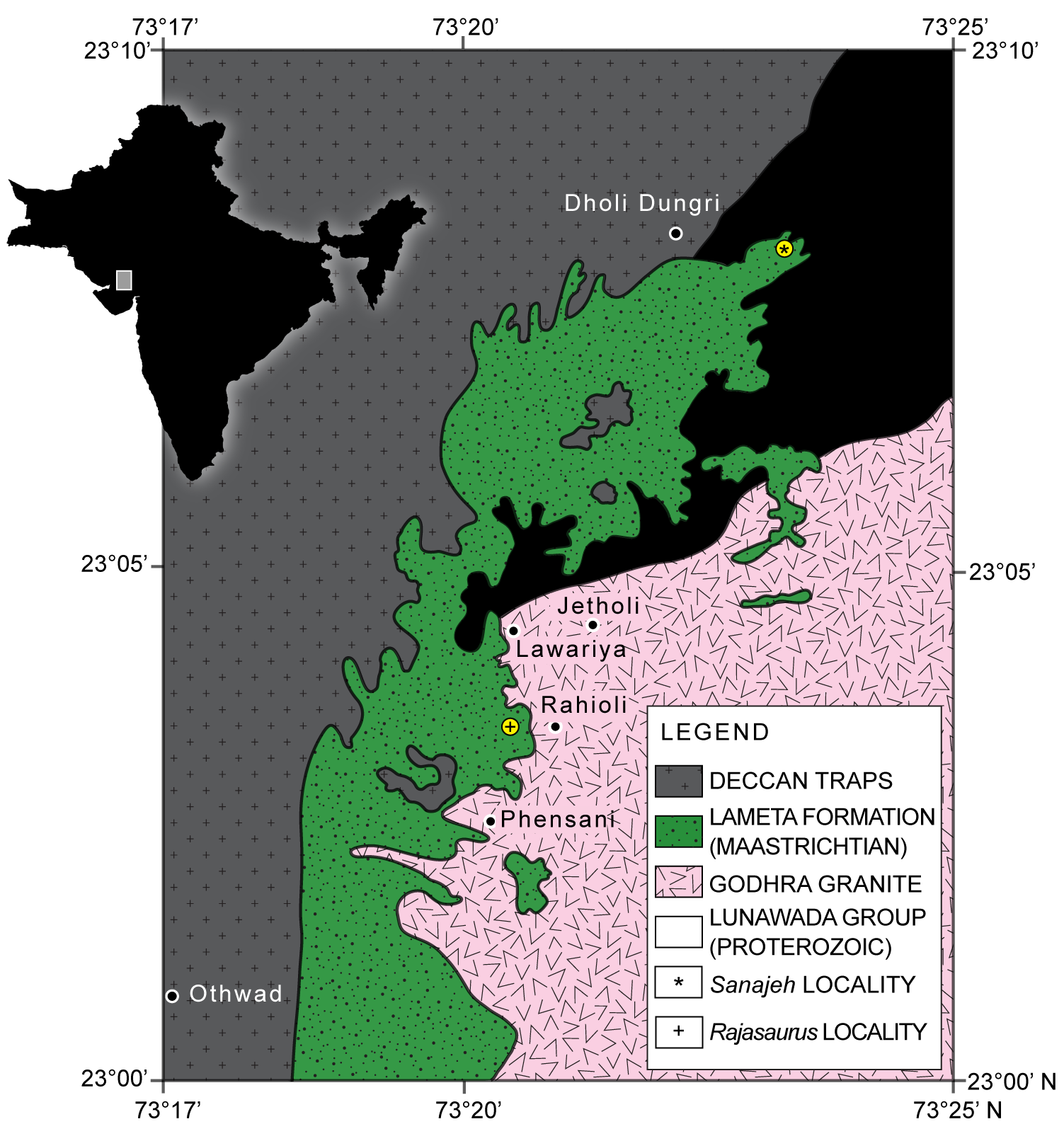

Supplement: Figure S1 — Geological map of rocks cropping out near the Dholi Dungri site in Kheda District, Gujarat (western India). Drafted by DMM. (0.58 MB TIF) [file pbio.1000322.s001.tif]

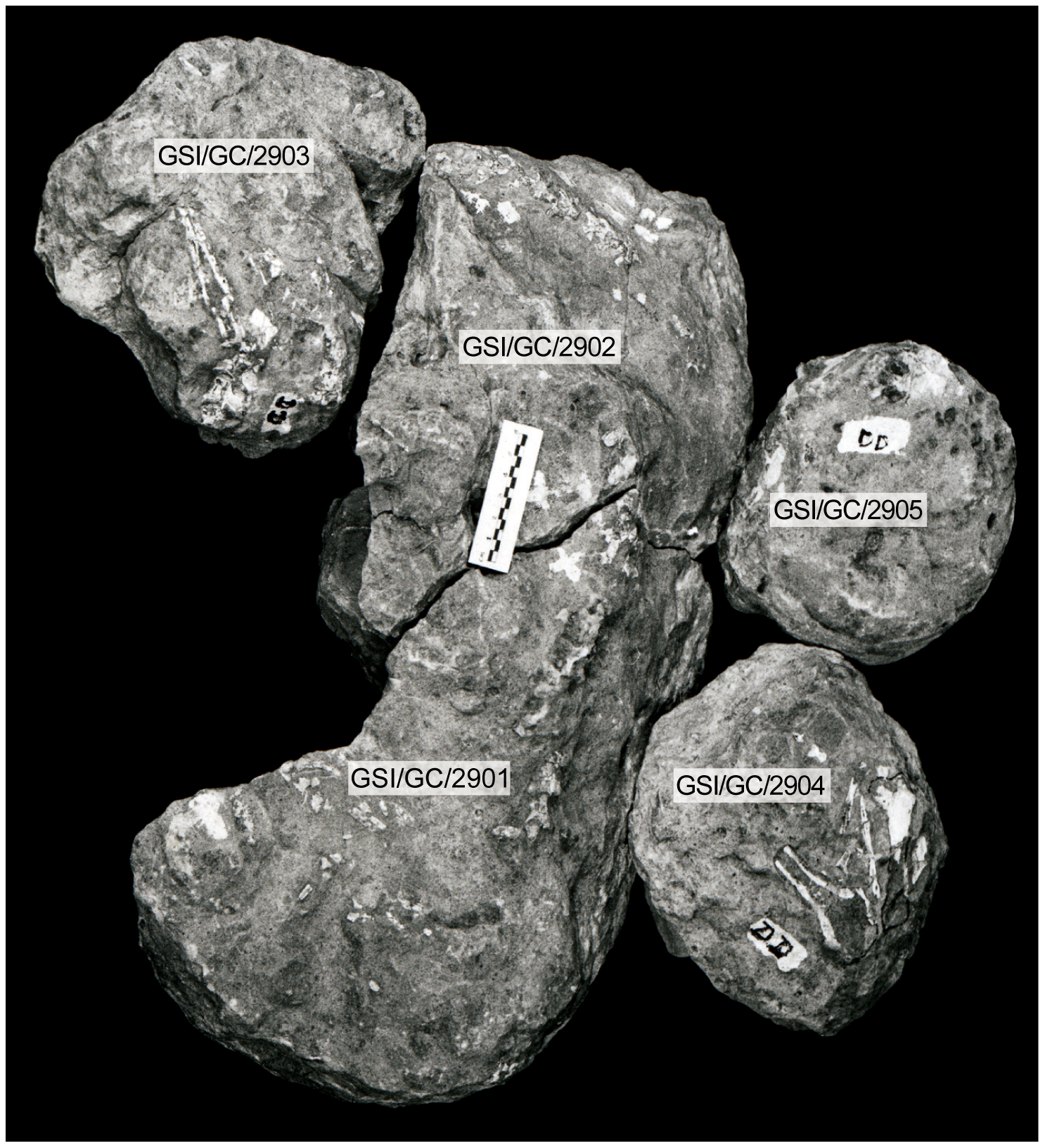

Supplement: Figure S2 — Snake-egg-hatchling blocks collected at Dholi Dungri, Gujarat State, India. This is a reproduction of a plate from [6], showing the initial state prior to preparation. Compare to Figure S3. Gray boxes indicate field numbers assigned to blocks. Scale is in centimeters. (3.43 MB TIF) [file pbio.1000322.s002.tif]

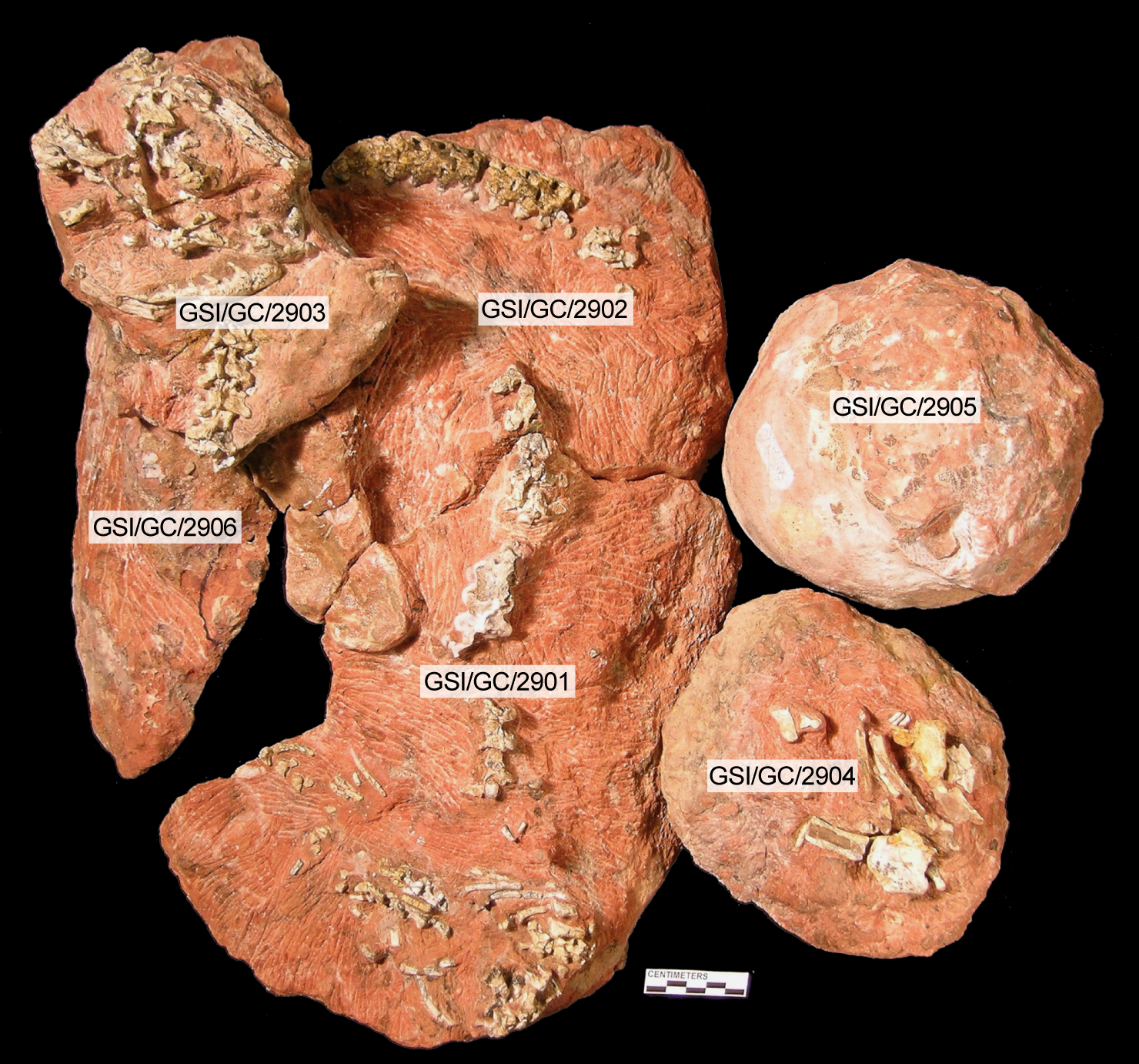

Supplement: Figure S3 — Fully prepared snake-egg-hatchling blocks. Note addition of the “Gandhinagar block” (GSI/GS/2906) and the different orientation and position of the cranial block (GSI/GC/2903). Scale is in centimeters. (4.70 MB TIF) [file pbio.1000322.s003.tif]

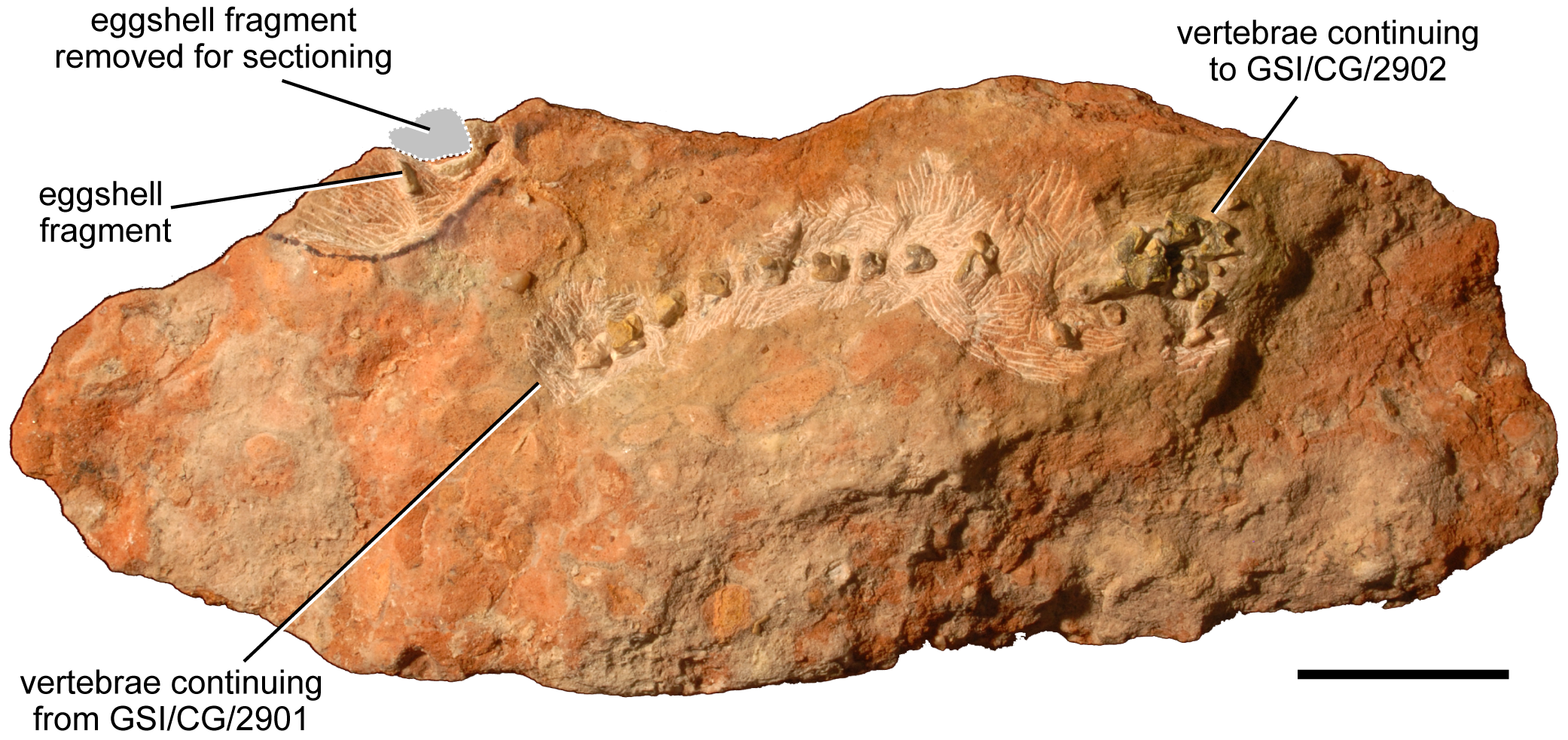

Supplement: Figure S4 — “Gandhinagar” block (GSI/GC/2906). This block preserves fragments of the crushed Megaloolithus egg and a chain of Sanajeh vertebrae connecting the series on blocks GSI/GC/2901 and GSI/GC/2902. This image shows the underside of the block shown in Figure S3. Scale equals 5 cm. (2.84 MB TIF) [file pbio.1000322.s004.tif]

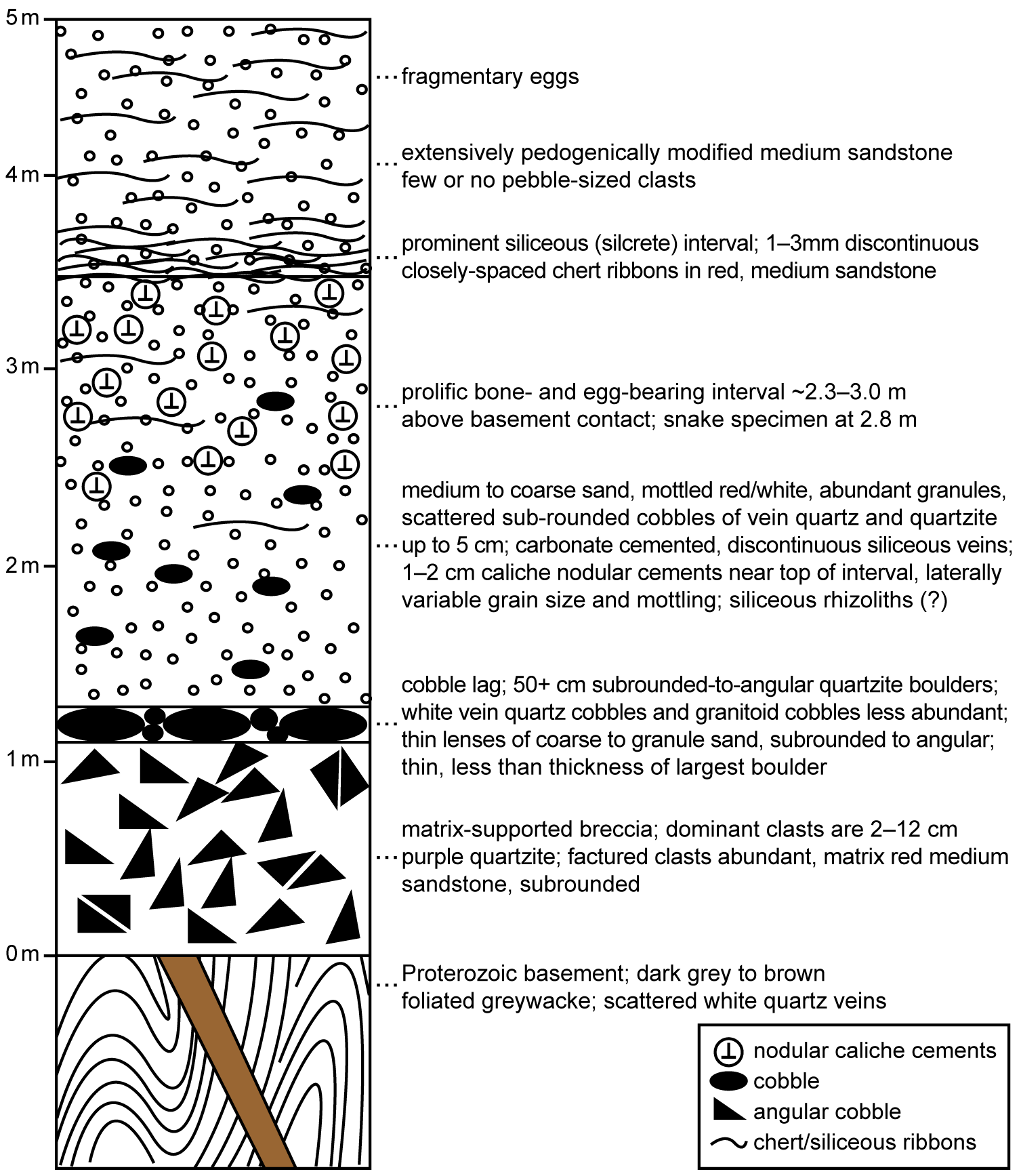

Supplement: Figure S5 — Stratigraphic column for Dholi Dungri. Section base is at 23° 07.754′ N, 73° 22.544′ E; terminus is at 23° 07.818′ N, 73° 22.544′ E. All unit contacts, with the exception of the boulder lag and Precambrian basement, are gradational. Lateral variability not reflected in this transect. Drafted by SEP. (0.38 MB TIF) [file pbio.1000322.s005.tif]

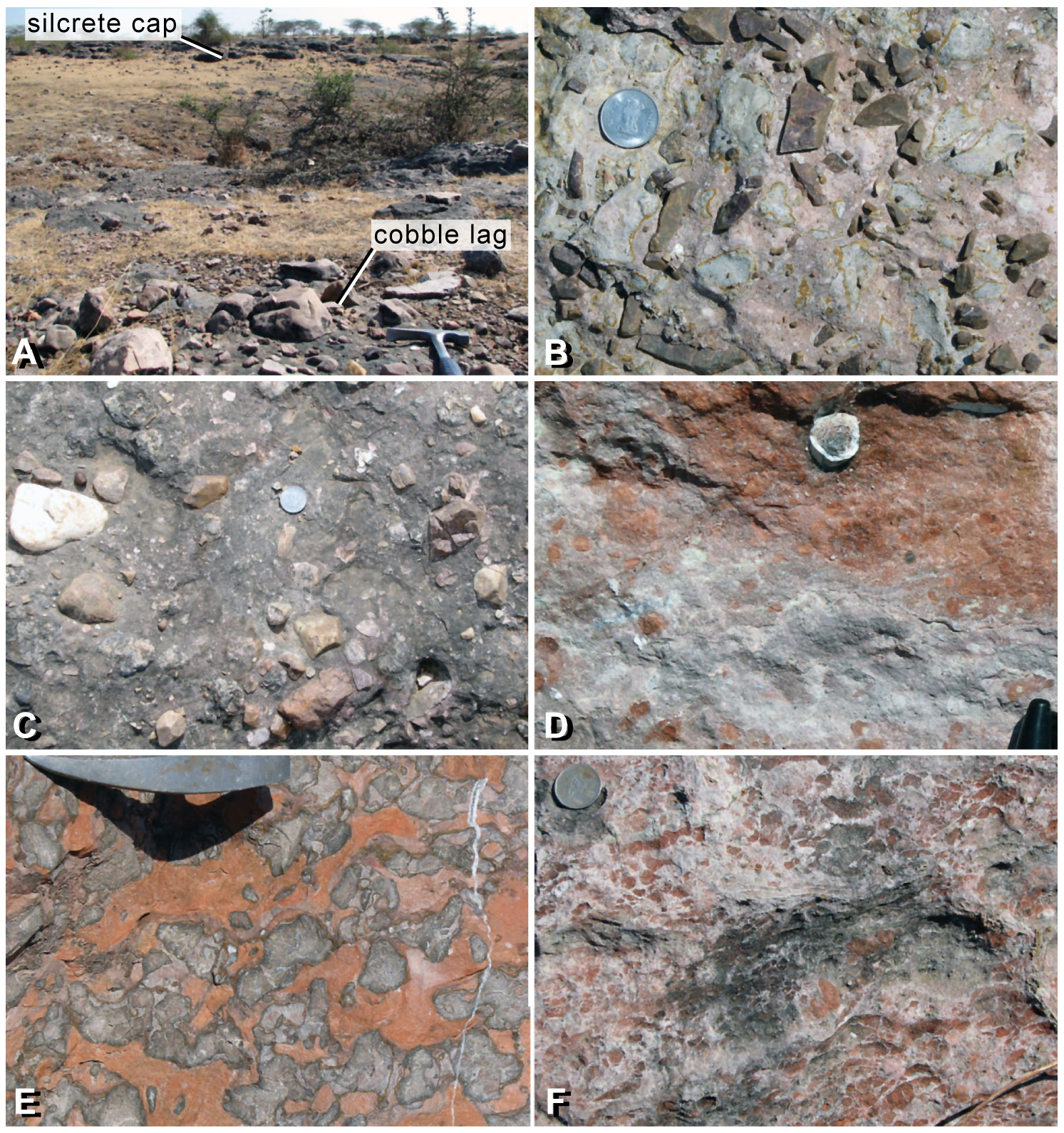

Supplement: Figure S6 — Stratigraphic and petrologic examples of the Lameta Formation at Dholi Dungri. (A) Overview of section near Sanajeh discovery site. Cobble lag at base of photo represents an ephemeral Maastrichtian drainage (see Figure S5). Resistant bed at top of slope is in silcrete interval near top of section. (B) Base of section, above Proterozoic basement. Carbonate- and silica-cemented, poorly sorted sand with angular quartzite clasts. (C-D) Fossil-bearing interval. (C) Carbonate- and silica-cemented, poorly sorted sand with subrounded quartzite and vein-quartz clasts. (D) cross-section showing bone fragment (top center of image). (E) Near top of section. Pedogenic fabric characteristic of nodular caliche interval (see Figure S5). (F) Silcrete interval. Discontinuous, resistant veins are composed of silica cements. The Lameta Formation at Dholi Dungri has been extensively diagenetically modified by silcrete and calcrete pedogenesis, but there is evidence for episodic sedimentation near a paleotopographic bedrock high. It is possible that this sedimentation resulted in the preservation of the snake-nest association. Rupee coins in (B, C, and F) are 2.5 cm in diameter. (4.75 MB TIF) [file pbio.1000322.s006.tif]

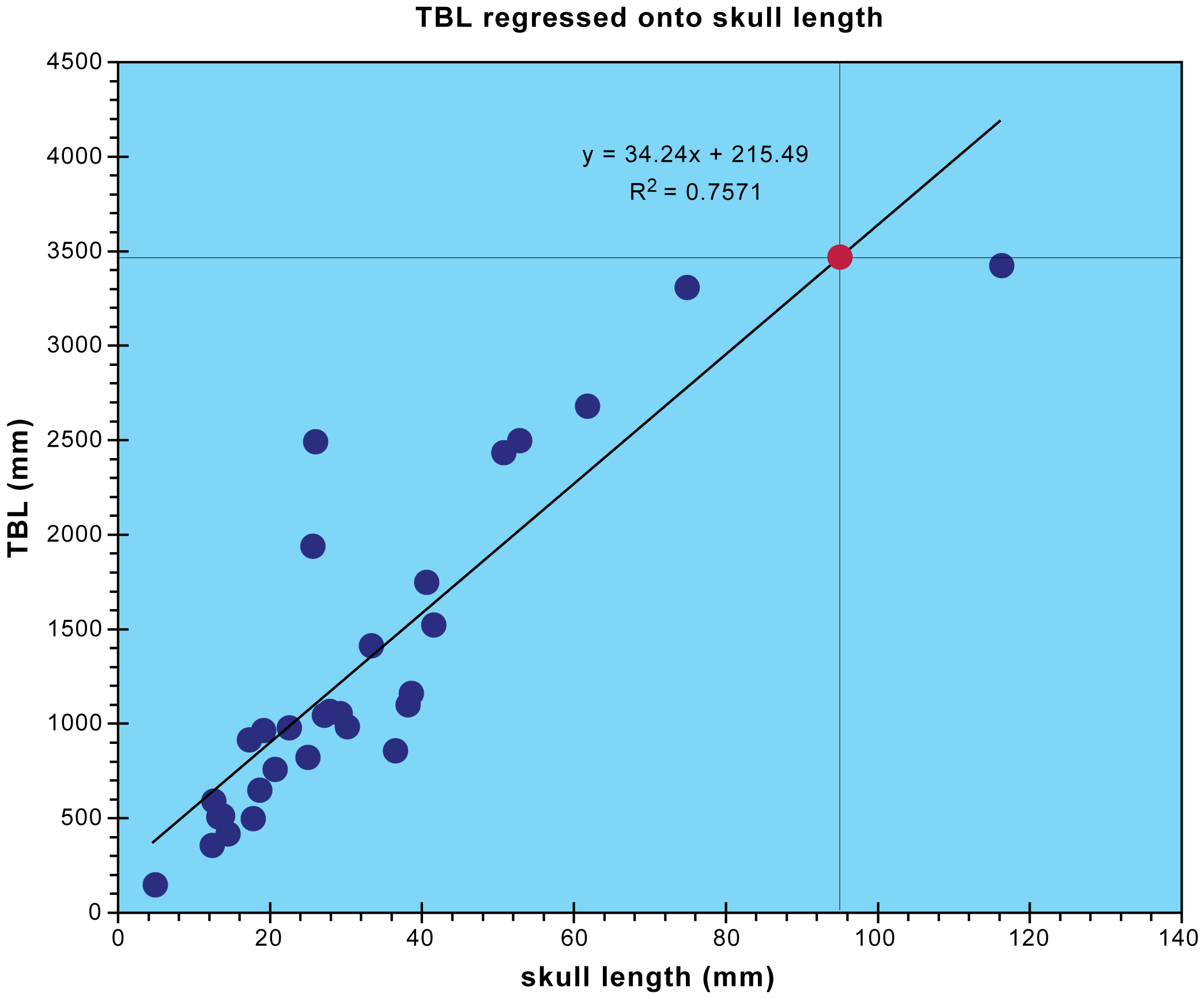

Supplement: Figure S7 — Body length estimate for S. indicus . An estimated skull length of 95 mm indicates a total body length (TBL) of 3.46 m. (0.19 MB TIF) [file pbio.1000322.s007.tif]

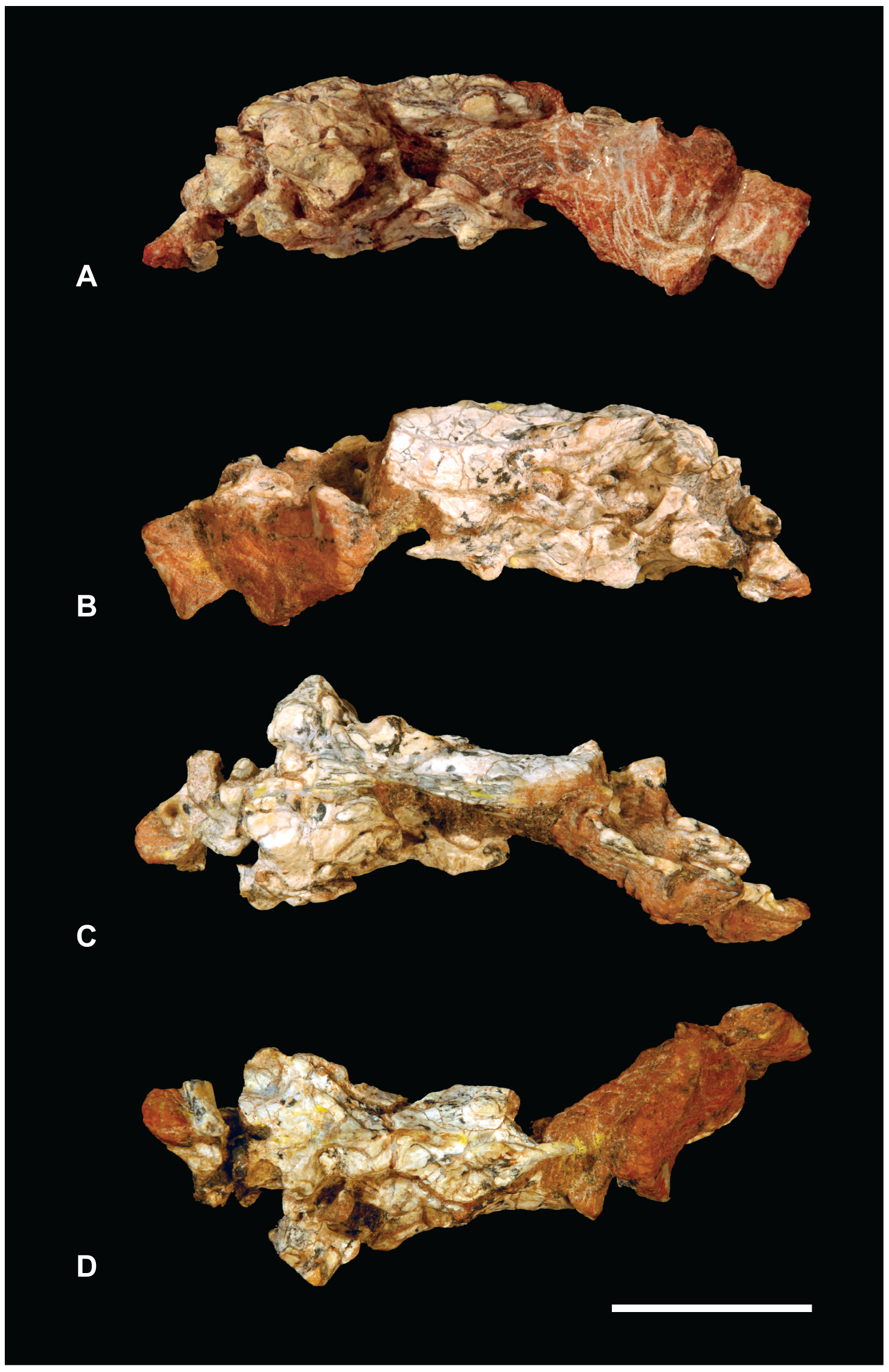

Supplement: Figure S8 — Braincase and skull roof S. indicus . Photographs in right lateral (A), left lateral (B), dorsal (C), and ventral (D) views. Scale equals 5 cm. (2.76 MB TIF) [file pbio.1000322.s008.tif]

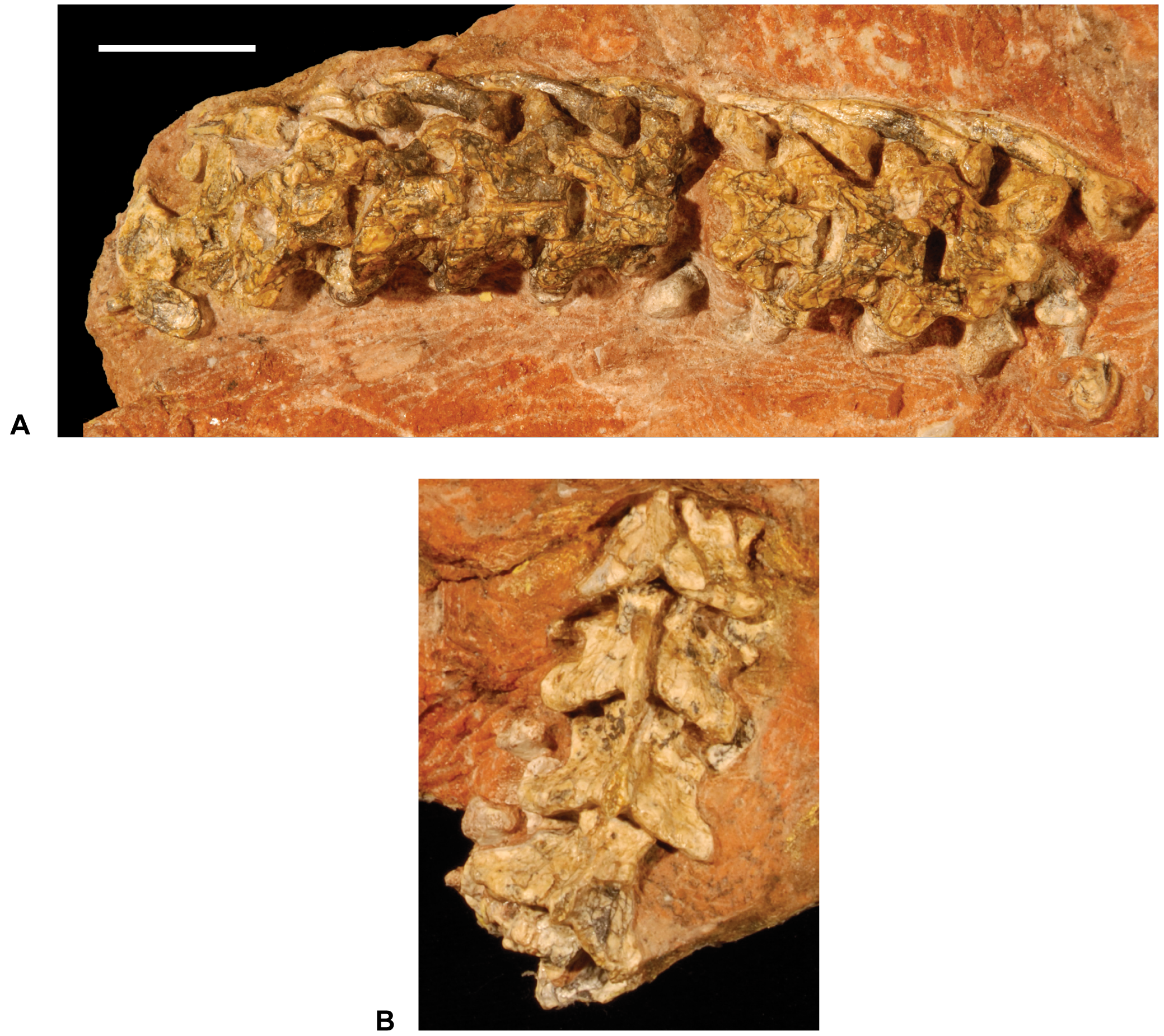

Supplement: Figure S9 — Articulated vertebrae of S. indicus . Photographs of vertebrae on block GSI/GC/2902 (A) and block GSI/GC/2903 (B) in dorsal view. Scale equals 2 cm for both images. (4.44 MB TIF) [file pbio.1000322.s009.tif]

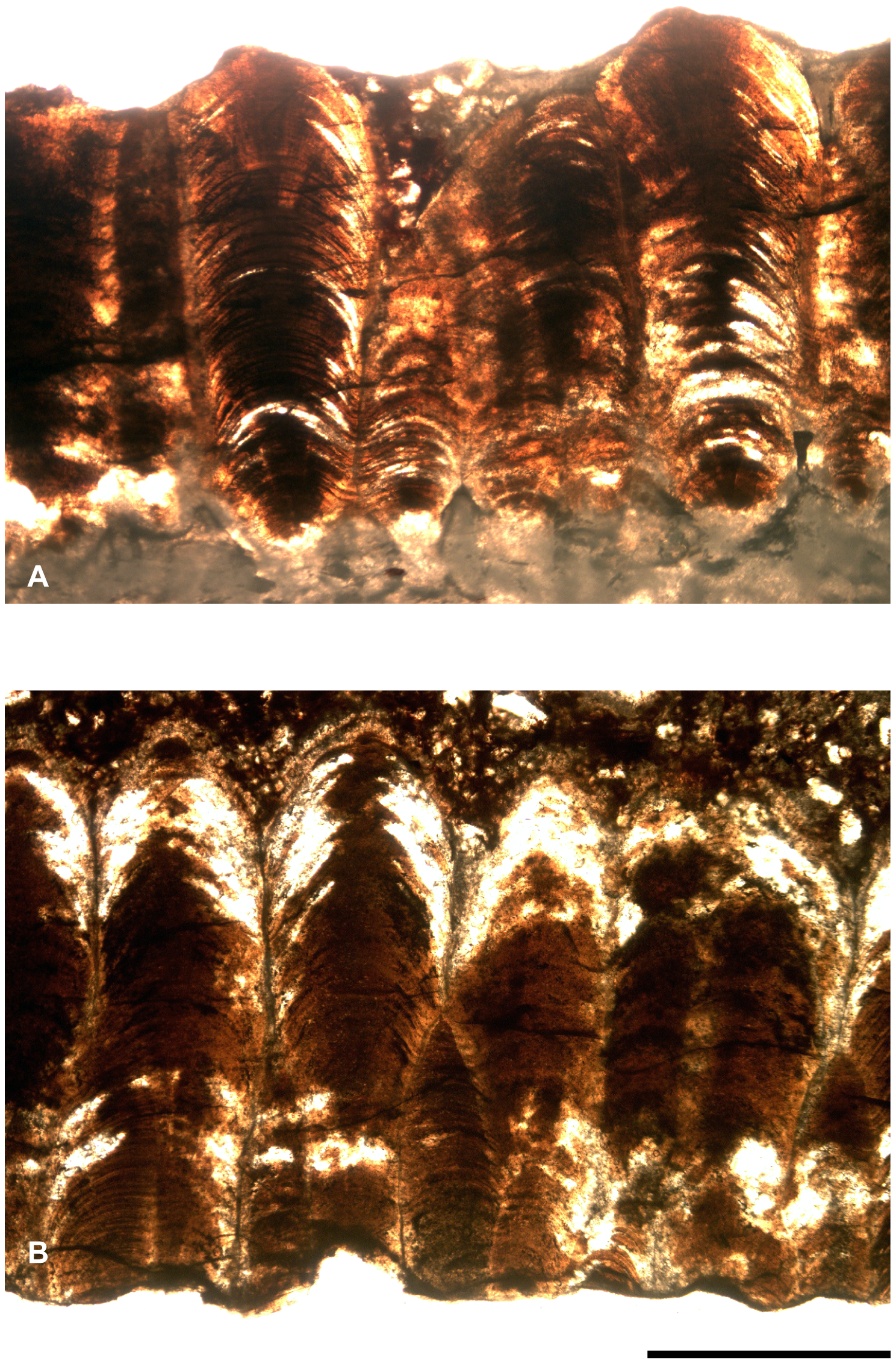

Supplement: Figure S10 — Megaloolithus eggshell histology. Thin-sections of uncrushed (A) and crushed (B) eggshell from blocks GSI/GC/2906 and GSI/GC/2905, respectively. Scale equals 1 mm. (6.69 MB TIF) [file pbio.1000322.s010.tif]

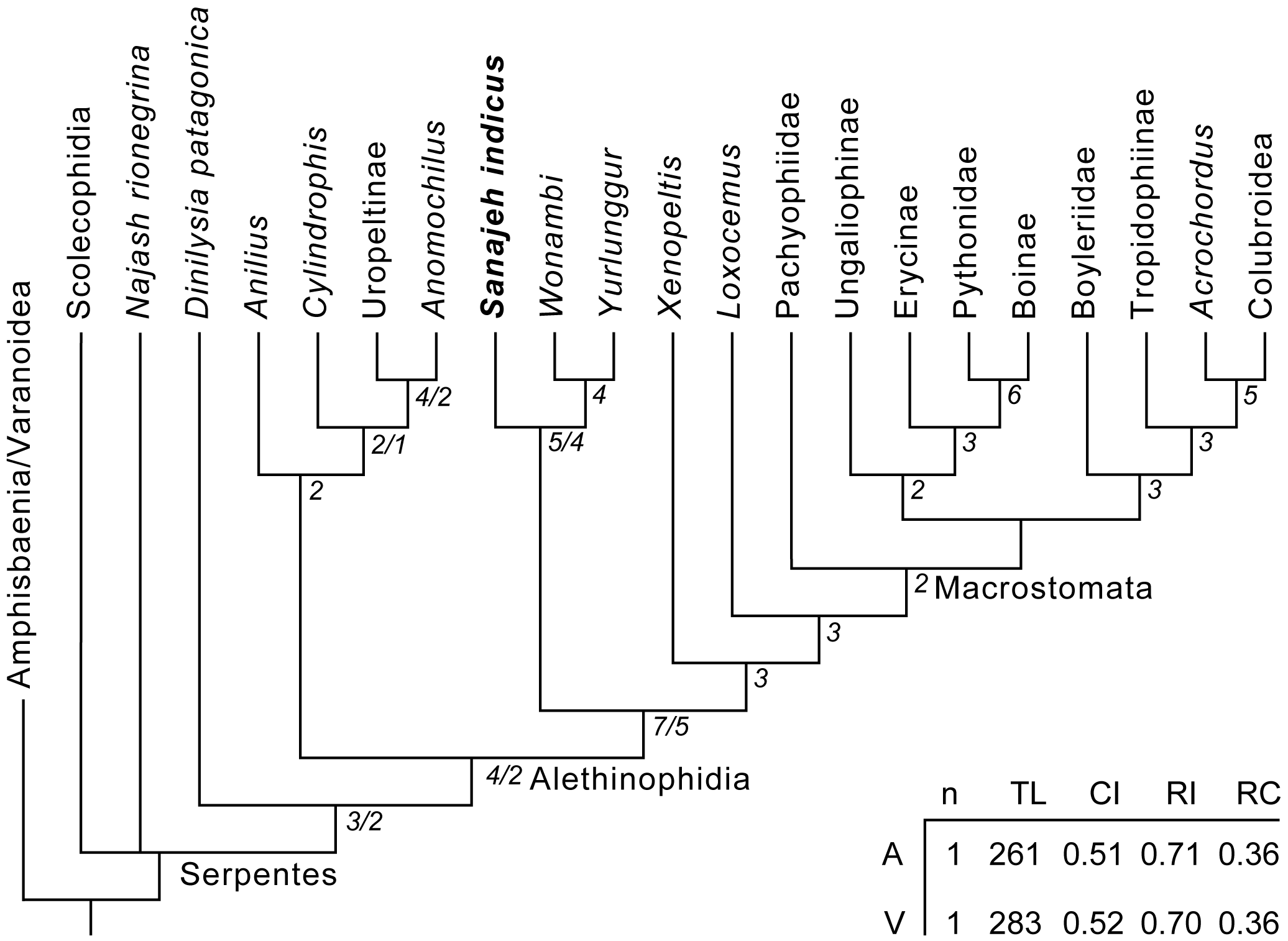

Supplement: Figure S11 — Consensus of the single most parsimonious trees derived from analyses employing Amphisbaenia and Varanoidea as outgroups. Topologies were identical except for the position of Najash relative to Scolecophidia and Dinilysia. Numbers at nodes indicate decay values greater than 1; where decay indices differ between analyses, both are reported (separated by a “/”). Trees rooted with Amphisbaenia have stronger support at basal nodes. Tree statistics are shown at lower right; n, number of trees; TL, treelength; CI, consistency index; RI, retention index; RC, rescaled consistency index; A, Amphisbaenia; V, Varanoidea. (0.23 MB TIF) [file pbio.1000322.s011.tif]

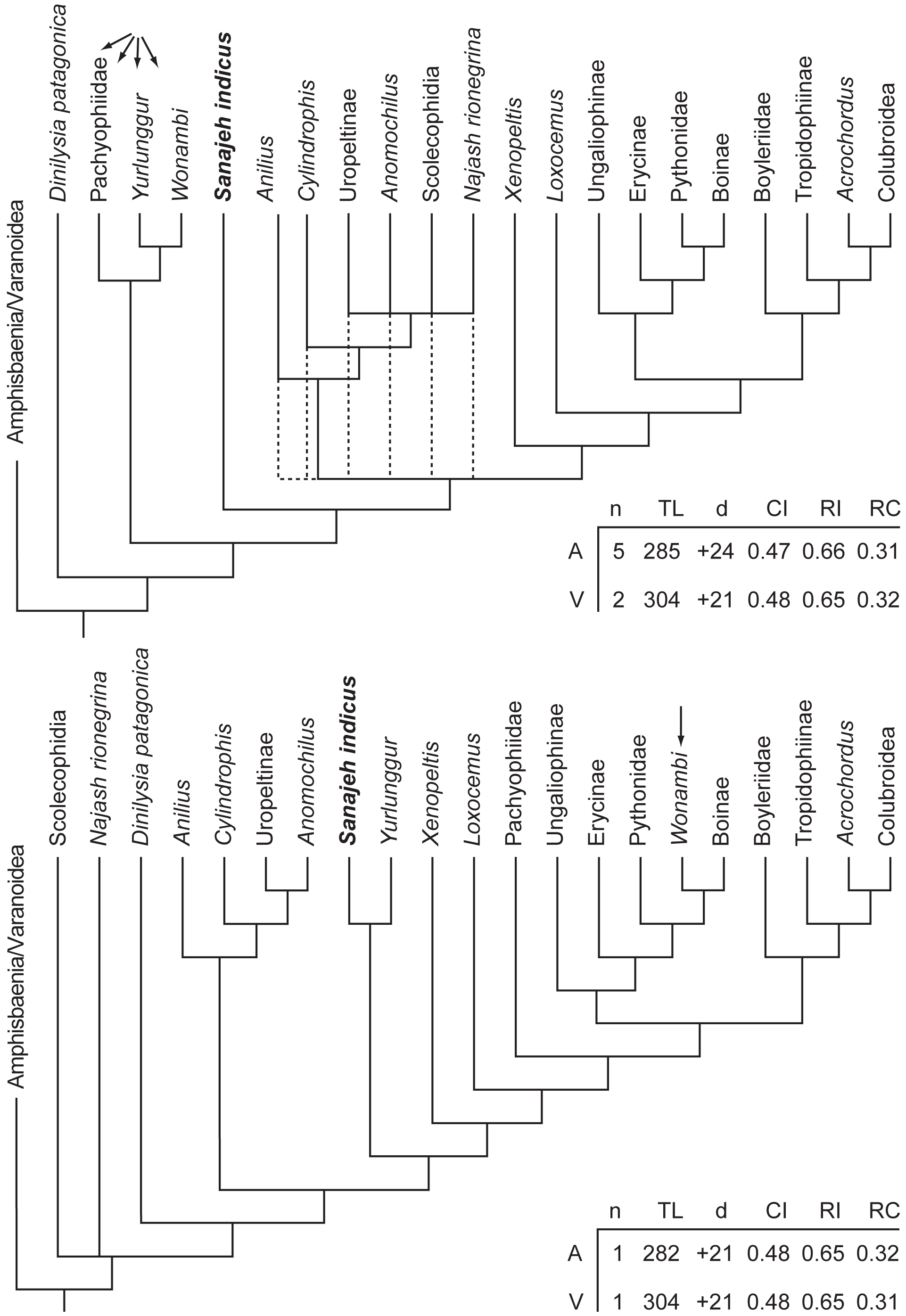

Supplement: Figure S12 — Constraint trees. Top, basal positions of Wonambi, Yurlunggur, Dinilysia, and Pachyophiidae were fixed at base of tree (but with no specified relationship to one another); bottom, a sister-taxon relationship between Wonambi and Boinae was fixed. Constrained taxa are indicated with arrows. Dashed lines in top cladogram indicate unresolved nodes in strict consensus of five trees rooted by Amphisbaenia. Tree statistics are shown in boxes at lower right; abbreviations as in Figure S11, except: d, parsimony debt under topological constraints. (0.36 MB TIF) [file pbio.1000322.s012.tif]
