# Supplementary material for: Predation upon Hatchling Dinosaurs by a New Snake from the Late Cretaceous of India
Source: PLoS Biol. 2010 Mar 2;8(3):e1000322. doi: 10.1371/journal.pbio.1000322 (PMC2830453; doi:10.1371/journal.pbio.1000322)
Supplement: Text S1 — History of the discovery. (0.03 MB DOC) [file pbio.1000322.s013.doc]

**TEXT S1. HISTORY OF THE DISCOVERY**

The first dinosaur nesting grounds in western India were discovered in exposures of the Lameta Formation near the villages of Rahioli, Balasinor, and Dholi Dungri (Figure S1) by the Geological Survey of India (GSI) in the early 1980s [1–3]. At Rahioli, sauropod and theropod teeth [4] and bones [5–7] were discovered in calcareous sandstone and conglomeratic levels underlying the egg-bearing level. There, a large bone bed containing both theropod and sauropod bones was excavated and mapped during 1982–4, and a subset of these bones were later described as *Rajasaurus narmadensis* [8]. Further excavations near this locality produced additional sauropod and theropod remains [9]. In 1986, one of us (DMM) discovered a block preserving eggs and bones near the village of Dholi Dungri that were then the only such association known from India. The initial descriptive paper [6], written well before the specimen was prepared (Figure S2), interpreted the specimen as a juvenile sauropod dinosaur preserved inside a nest. Although identification of sauropod egg and hatchling sauropod rib, scapula, and humerus was correct, the vertebrae were incorrectly identified. S. L. Jain [10] was the first to correctly identify the vertebrae preserved on the main block as pertaining to a snake, an observation that went largely unnoticed and was never followed by detailed study. In 2001, one of us (JAW) reexamined the specimen and independently arrived at the same conclusion as Jain made 12 years earlier. Further study in the GSI collections by DMM and JAW uncovered a block that was collected with the original specimen but was never described and, as a consequence, had been dissociated from it (see below). In 2004, the specimen was then fully prepared at the University of Michigan Museum of Paleontology (Figure S3). In 2007, the authors collected geological and paleontological data at Dholi Dungri, including the discovery of multiple associations between the snake *Sanajeh indicus* and sauropod eggs.

The holotype of *Sanajeh indicus*, the *Megaloolithus dhoridungriensis* eggs, and the partial sauropod hatchling were collected by the GSI in six blocks, only five of which were reported in the brief original description [6] (Figures S2, S3). The sixth block became dissociated from the other blocks and was lost until 2004, when it was rediscovered in collections of the GSI (Western Region) in Gandhinagar by JAW and DMM. This new block has a snap-fit onto the two main blocks (GSI/GC/2901,2902) and preserves a chain of vertebrae on its underside that bridges the series of vertebrae present on the adjoining blocks, completing a loop around the crushed egg (Figure S4). The rediscovered “Gandhinagar” block contains remnants of the crushed egg, which are also preserved on the two adjoining main blocks. The field number and description of the six blocks are:

| field number | description |
| --- | --- |
| GSI/GC/2901 | *Sanajeh* postcranium block containing part of the crushed *Megaloolithus dhoridungriensis* egg |
| GSI/GC/2902 | *Sanajeh* postcranium block containing part of the crushed *Megaloolithus dhoridungriensis* egg |
| GSI/GC/2903 | *Sanajeh* skull block |
| GSI/GC/2904 | sauropod hatchling block |
| GSI/GC/2905 | complete, uncrushed *Megaloolithus dhoridungriensis* egg |
| GSI/GC/2906 | “Gandhinagar block” containing a chain of *Sanajeh* vertebrae and fragments of the crushed *Megaloolithus dhoridungriensis* egg |

The “Gandhinagar” block originally was given the same field number as the sauropod hatchling block (GSI/GC/2904)—which may explain why its absence in the original description was not missed. We assigned the “Gandhinagar block” number GSI/GC/2906 to avoid further confusion. The skull block (GSI/GC/2903) fits atop a saddle formed by the “Gandhinagar block” and main block GSI/GC/2902 (Figure S3). Although this positioning of skull block (GSI/GC/2903) differs from that in the original publication (Figure S2, [6]), eggshell fragments on the underside of the skull block (GSI/GC/2903) and the snap-fit between blocks confirm its position atop the crushed egg. The *Megaloolithus dhoridungriensis* egg (GSI/GC/2905) and the juvenile block (GSI/GC/2904) do not have snap-fits onto the main blocks, so we have reconstructed their position based on the original publication [6] and subsequent investigation at the site. A third, uncrushed egg that was not reported in the original description is still in place at the site. This egg, which has been hemisected, was positioned near the skull block.

**Literature Cited in TEXT S1**

1. Mohabey DM (1982) On the occurrence of dinosaurian fossil eggs from infratrappean limestone, Kheda District, Gujarat. Curr Science 52: 1194–1195.

Mohabey DM (1984) The study of dinosaurian eggs from infratrappean limestone in Kheda District, India. J Geol Soc India 25: 329–337.

Srivastava S, Mohabey DM, Sahni A, Pant SC (1986) Upper Cretaceous dinosaur egg clutches from Kheda District (Gujarat, India). Palaeontographica (Abt A) 193: 219–233.

Mathur UB, Srivastava S (1987) Dinosaur teeth from Lameta Group (Upper Cretaceous) of Kheda District, Gujarat. J Geol Soc India 29: 554–566.

Mathur UB, Pant SB (1986) Sauropod dinosaur humeri from Lameta Group (Upper Cretaceous-?Palaeocene) of Kheda District, Gujarat. J Palaeontol Soc India 31: 22–25.

Mohabey DM (1987) Juvenile sauropod dinosaur from Upper Cretaceous Lameta Formation of Panchmahals District, Gujarat, India. J Geol Soc India 30: 210–216.

Mohabey DM (1989) The braincase of a dinosaur from the Late Cretaceous Lameta Formation, Kheda District, Gujarat, Western India. Indian J Earth Sci 16: 132–135.

Wilson JA, Sereno PC, Srivastava S, Bhatt DK, Khosla A et al. (2003) A new abelisaurid (Dinosauria, Theropoda) from the Lameta Formation (Cretaceous, Maastrichtian) of India. Contrib Mus Paleontol Univ Michigan 31: 1–42.

Chatterjee S, Rudra DK (1996) KT events in India: impact, rifting, volcanism and dinosaur extinction. Mem Queensland Mus 39: 489–532.

Jain SL (1989) Recent dinosaur discoveries in India, including eggshells, nests and coprolites. In: Gillette D, Lockley M, editors. Dinosaur Tracks and Traces. New York: Cambridge University Press. pp. 99–108.
