# Supplementary material for: Predation upon Hatchling Dinosaurs by a New Snake from the Late Cretaceous of India
Source: PLoS Biol. 2010 Mar 2;8(3):e1000322. doi: 10.1371/journal.pbio.1000322 (PMC2830453; doi:10.1371/journal.pbio.1000322)
Supplement: Text S2 — Geological setting. (0.03 MB DOC) [file pbio.1000322.s014.doc]

**TEXT S2. GEOLOGICAL SETTING**

The Late Cretaceous (Maastrichtian) Lameta Formation of central and western India records a diverse suite of terrestrial environments, including low-gradient fluvio-laustrine flood plains, braided rivers and soil-forming uplands [1]. A paleomagnetically derived paleolatitude of approximately 29 degrees south and abundant sedimentological and diagenetic evidence indicate that the Lameta Formation formed in a semi-arid, tropical wet-dry climate [2]. Although such environmental characteristics have been well documented through a number of excellent regional field studies, the uniqueness of the Lameta Formation within the broader geological context of the global Mesozoic sedimentary record, which is dominated by sediment accumulated in tectonically active sedimentary basins and in marginal marine settings, has received little attention. The Lameta Formation represents the surface of a mostly erosional terrestrial setting with substantial regional and local paleotopographic relief. Were it not for the emplacement of the overlying flood basalts of the Deccan Traps, which deeply buried Maastrichtian topography, it is unlikely that any of the environments present in the Lameta Formation would have survived to the present day. It is probably for this reason that the Lameta Formation has afforded such unique insights into Late Cretaceous terrestrial life [3].

Exposures of the Lameta Formation in Kheda District, Gujarat State, are thin (generally less than 8 m) and dominated by calcareous and siliceous quartzose sandstones, arkoses that are occasionally associated with red and green marl, and conglomerates that contain few or no primary sedimentary structures. Most of the coarse grained, immature sediments derive from immediately underlying and adjacent bedrock, which in this region consists of Proterozoic metasediments and intrusive granitic rocks. Nearly all of the Lameta Formation sediments at Dholi Dungri are heavily overprinted by pedogenic calcretes and silcretes, and pedogenic modification may have contributed to the paucity of visible primary sedimentary structures. A more detailed, regional synthesis of the diverse paloenvironments represented in Gujarat State will be accomplished in future work; here we provide an outline of the environmental conditions that are associated with the preservation of the Dholi Dungri specimen.

The snake-bearing interval at Dholi Dungri is characterized by a succession of facies that are unique both in the immediately adjacent area and in the broader region (Figures S5, S6). The two most notable characteristics at Dholi Dungri are: 1) a thin lag concentration of subrounded to subangular boulders of tan quartzite, up to 60 cm in size, with intercalated cobble and granule to medium sand lenses, and 2) articulated and associated vertebrate remains found together with eggs. Neither the boulder lag nor the articulated vertebrate remains can be traced over lateral distances greater than approximately 100 m, despite adequate exposure. We interpret the boulder lag and intercalated granule sands as representing a relatively high-gradient intermittently flowing fluvial channel segment that transported boulder-size clasts only short distances from immediately adjacent outcrops of Proterozoic quartzites. The channel itself overlies softer, more easily weathered phyllitic Proterozoic greywackes, but thick quartzite beds are clearly exposed within 500 m of the site.

Physical tracing of the Lameta-basement contact at Dholi Dungri demonstrates that the fossil site was located adjacent to two important, paleogeomorphic features: (1) a topographic high generated by folded Precambrian orthoquartzites within less resistant schistose graywackes, and (2) a small tributary drainage system. Mapping of the Lameta-Precambrian contact in the Dholi Dungri region demonstrates paleotopographic relief of over 14 m in less than 300 lateral meters. Further work is required to provide more detailed paleotopographic resolution.

Interpretation of the poorly sorted, cobble-bearing medium to coarse sand interval that contains articulated vertebrates and sauropod nests is confounded by pedogenesis and the lack of any primary sedimentary structures visible on outcrop. Storm-induced debris flows, transported short distances from adjacent paleotopographic highs or within the small paleochannel itself, are likely candidates for rapid sedimentation events, particularly in the subtropical wet-dry environment known to have characterized this environment in the late Maastrichtian. Debris flows, subsequently modified by bioturbation (e.g., root formation, nesting sites) and pedogenesis, would lack clearly defined primary sedimentary structures, would be limited in areal extent, and would be expected given the inferred paleotopography present in the immediately adjacent area. Debris flows would also provide a sufficiently rapid pulse to preserve the observed ecological associations, such as those suggested by the Dholi Dungri specimen. Although we cannot as yet precisely determine the exact taphonomic scenario that resulted in the preservation of the snake-hatchling-egg association, the unique position of the site relative to the paleotopography created by differential weathering of Precambrian metasediments is compelling evidence for unusual sedimentation events in an otherwise degradational, soil-forming environment.

**Literature Cited in TEXT S2**

Mohabey DM, Udhoji SG, Verma KK (1993) Palaeontological and sedimentological observations on non-marine Lameta Formation (Upper Cretaceous) of Maharashtra, India: their palaeoecological and palaeoenvironmental significance. Palaeogeogr Palaeoclimat Palaeoecol 105: 83–94.

Tandon SK, Sood A, Andrews JE, Dennis PF (1995) Palaeoenvironments of the dinosaur-bearing Lameta Beds (Maastrichtian), Narmada Valley, Central India. Palaeogeogr Palaeoclimat Palaeoecol 117: 153–184.

Mohabey DM (1996) Depositional environments of Lameta Formation (Late Cretaceous) of Nand-Dongargaon inland basin, Maharashtra: the fossil and lithological evidence. Mem Geol Surv India 377: 363–386.
