# Supplementary material for: Predation upon Hatchling Dinosaurs by a New Snake from the Late Cretaceous of India
Source: PLoS Biol. 2010 Mar 2;8(3):e1000322. doi: 10.1371/journal.pbio.1000322 (PMC2830453; doi:10.1371/journal.pbio.1000322)
Supplement: Text S3 — Body size estimate for S. indicus . (0.02 MB DOC) [file pbio.1000322.s015.doc]

**TEXT S3. BODY SIZE ESTIMATE FOR *SANAJEH INDICUS***

We estimated body size in the snake *Sanajeh indicus* by constructing a regression model of total body length onto skull length for crown-group snakes (Figure S7). We sampled taxa at all levels of snake phylogeny to construct the model (see examined specimens, Text S6). We measured skull length in dorsal view along the midline, from the anterior tip of the premaxilla to the posterior margin of the otooccipital above the foramen magnum. This measure records skull length without incorporating the posterior elongation and displacement of the mandibular suspensorium that is present in macrostomatans.

Regression produced a significant positive relationship (R=0.76, *p* <0.001) expressed by the equation y = 34.2x + 215.4. We estimated skull length in *Sanajeh* to be 95 mm by summing the midline length of the braincase to the approximate anterior margin of the frontal articulation, the length of the maxilla to the level of the dorsal process, and the estimated length of the premaxilla. Incorporation into the regression equation produces an estimated total body length of 3.46 m (Figure S7).
