# Supplementary material for: Predation upon Hatchling Dinosaurs by a New Snake from the Late Cretaceous of India
Source: PLoS Biol. 2010 Mar 2;8(3):e1000322. doi: 10.1371/journal.pbio.1000322 (PMC2830453; doi:10.1371/journal.pbio.1000322)
Supplement: Text S4 — Additional anatomical description. (0.06 MB DOC) [file pbio.1000322.s016.doc]

**TEXT S4. ADDITIONAL ANATOMICAL DESCRIPTION**

The skull of *Sanajeh indicus* preserves right and left maxillae, right palatine, fragmentary frontals, a nearly complete, articulated braincase (including partial parietal, prootics, otooccipitals, supraoccipital, supratemporal, basioccipital, and parabasisphenoid; Figure S8), and right and left mandibles (including a well-preserved right dentary).

**Maxilla**—The maxillae are represented by a nearly complete right element that is visible in dorsal view and a distal portion of the left maxilla that is visible in lateral view. The maxilla is elongate, with a short narial region measuring approximately 30% of its total length. The anterior process is medially recurved, as it is in most other snakes. A small medial shelf is present, although slightly distorted, on the anterior process, as in anilioids, *Wonambi*, *Yurlunggur*, and *Nanowana* [1–3]. The dorsal process of the maxilla is taller than in *Wonambi*, *Yurlunggur*, and more derived taxa, and it is similar in size and shape to *Anilius* and *Cylindrophis*. A fragment of the right prefrontal is preserved in articulation with the dorsal process of the maxilla and forms the lateral walls of the lacrimal duct. The posterior margin of the maxilla is elongate, extending far posterior beyond the level of the orbit, as in *Wonambi* and *Yurlunggur* [2,3]. The posterior process possesses a shallow trough on the dorsal surface that receives the ectopterygoid.

**Frontal**—Two incomplete frontals are preserved and visible in posterior and posteromedial views (Figure S8). They are elongate, narrow elements and possess a ventromedially concave lateral pillar forming a shallow groove for the olfactory tract.

**Parietal**—The left side of the parietal along the length of the element and the right posterior region are preserved. The parietal is more elongate than in derived snakes, resembling the condition in anilioids. In dorsal view, the anterior margin consists of a poorly preserved but deep embayment to receive the frontals, as in anilioids, *Xenopeltis* and *Yurlunggur*. The base of a large sagittal crest is preserved along the midline of the element, posterior to the frontal articulation. Posteriorly, the parietal terminates laterally as an expanded, triangular supratemporal process that meets the posterior margin of the prootic and the anterodorsal margin of the otooccipital to form a large paraoccipital extension. The posterior margin of the parietal is slightly raised, forming the anterior limit of a shallow articular facet for the overlapping supratemporal. The suture of the parietal with the supraoccipital is poorly preserved, but appears have a median notch and posteriorly convex lateral margins. In lateral view, the parietal forms the anterior margin of the trigeminal foramen (CN V). The lateral surface of the element is dorsally expanded above the trigeminal foramen and otic region, more so than in most other taxa.

**Supraoccipital**—The supraoccipital is anteroposteriorly short. The anterior margin is poorly preserved, but it appears to have a slightly triangular apex that articulates with the parietal. Laterally, the supraoccipital extends onto the dorsal surface of the paraoccipital region of the braincase, terminating at the articulation of the parietal, prootic, and otooccipital. The posterior margin of the supraocciptal has an elongate spine that divides the anterior portion of the contact between the otooccipitals. The periosteal surface of the element is too poorly preserved to determine the presence or absence of transverse or sagittal crests.

**Supratemporal**—The right supratemporal was disarticulated but preserved near the posterolateral margin of the skull, overlying the right juxtastapedial recess. The anterior margins of the element are squared off, with horizontal dorsal and ventral margins and a vertical anterior margin. The medial margin of the element is strongly recurved in a fashion mirroring the convex dorsal surface of the posterior braincase. The posterolateral margin is an elongate, flared, free-ending process that extended slightly beyond the posterior otooccipital margin. This morphology is most similar to *Xenopeltis* among snakes. The ventral margin of the supratemporal is beveled along its length, forming a long articular facet for the quadrate. The length of the articular surface and its ventral orientation indicates that the quadrate was short and wide, probably retaining a large suprastapedial process.

**Prootic**—The anterodorsal margin of the right prootic is broken. The left prootic is dorsally displaced and crushed along the bridge that forms the posterior margin of the trigeminal foramen and anterior margin of the fenestra ovalis. The anterior process of the prootic forms the dorsal margin of the trigeminal foramen and is similar to the processes in *Wonambi* and *Yurlunggur* [3,4]. The process appears to contact the parietal as a simple articulation. The trigeminal foramen lacks a ventral prootic flange unlike *Yurlunggur*, *Wonambi*, and extant alethinophidians. The dorsal and ventral margins of the trigeminal foramen are widely separated anteriorly by the posterior margin of the parietal.

Posteriorly, the dorsal margin of the prootic is elongate and forms the anterodorsal margin of the juxtastapedial recess. The process is laterally expanded to meet the broad paraoccipital process of the otooccipital. The lateral margin of the prootic between the trigeminal foramen and fenestra ovalis is crushed. An opening for the exit of CN VII is present, but the lateral surface of the element is too poorly preserved to determine if the opening is singular or divided. The crista prootica includes a ventrally deflected lip that is confluent with the arc of the opisthotic crista interfenestralis and short relative to the condition in other taxa (e.g., boids, pythonids, many colubroids), indicating that the stapedial footplate would be mostly exposed in lateral view. The ventral margin of the prootic is broadly triangular with a ventral apex that articulated with the parabasisphenoid and basioccipital.

**Otooccipital**—The otooccipital forms the posterior margin of the juxtastapedial recess, as preserved on the right side of the skull. The juxtastapedial recess is elongate and occupies the majority of the lateral surface of the posterior braincase, as it does in *Wonambi* and *Yurlunggur* [2,3]. Within the recess, the crista interfenestralis divides the anterior foramen ovale from the lateral opening of the recessus scalae tympani as exposed on left side of the skull. The crista interfenestralis is anteriorly concave and has a laterally expanded ventral margin that terminates in a large, elongate, and rectangular accessory process. This feature is also present in *Wonambi* [2], *Xenopeltis* [5], and more derived macrostomatans [6]. The recessus ampullaris is present along the posterodorsal margin of the foramen ovale, just medial to the crista interfenestralis. The posterior margin of the crista interfenestralis on the left side of the skull preserves a small notch corresponding to the position of the anterior margin of the recessus scalae tympani in *Wonambi* [4]. The remainder of the recessus scalae tympani is not preserved on the left side of the skull, and it is obscured by the displaced supratemporal on the right side. The crista tuberalis forms the posterior margin of the juxtastapedial recess, as preserved on the right side of the skull. The crista tuberalis is prominent, and it is posteroventrally angled in lateral view and ventrolaterally angled in posterior view. The ventral margin of the crista tuberalis terminates as a wide, flared lateral margin of the basioccipital posterolateral process. The crista tuberalis component of the process extends laterally beyond the level of the paraoccipital process. The processes in *Wonambi* and *Yurlunggur* [3] are similar to the condition in *Sanajeh*, in which the process extends posteriorly toward the level of the occipital condyle. The extreme development in all three taxa is unique relative to any other snake or outgroup.

In lateral view, the paraoccipital process overhangs the juxtastapedial recess. The process is broadly triangular in dorsal and posterior views, and is laterally extensive as in basal snakes. The element is relatively smaller in posterior view than the processes of *Wonambi* and *Yurlunggur*, and does not possess the wide, heavily interdigitated articular facet for the supratemporal seen in *Wonambi*. Instead, the articular facet is a simple, curved surface that is slightly recessed relative to the posterior margins of the parietal and supraoccipital.

The posterior margins of the otooccipitals are broken, but they extend well beyond the short supraoccipital, indicating that the elements exclude the supraoccipital from the dorsal margin of the foramen magnum.

**Basioccipital**—The basioccipital is nearly complete, lacking only its left lateral margin. The element is broadly subtriangular in ventral view and has a short, horizontal articulation with the prootic positioned at the level of the anterior margin of the juxtastapedial recess in lateral view of the skull. The anteroventral margin of the basioccipital bears a prominent sagittal crest that serves as the origin of m. protractor pterygoidei. The crest extends along approximately the first one-third of the basioccipital and continues anteriorly onto the parabasisphenoid. The sagittal crest terminates at a low, sinuous transverse crest that extends to the posterolateral margin of the basioccipital. This smaller crest was previously considered a synapomorphy of *Wonambi* + Boinae (”Basisphenoid-basioccipital suture smooth (0), transversely crested (1)” [7]; however, our study identifies a crest in multiple taxa. The anterolateral margin of the basioccipital articulates with the prootic in a straight-line suture. The crista interfenestralis accessory process abuts the lateral margin of the basioccipital just posterior to the prootic. The posterolateral process of the basioccipital (posterolateral flanges of [1]) is expanded so that only a small space separates it from the elongate occipital condyle. The suture between the basioccipital and the otooccipital is laterally concave, similar in shape but relatively more lateral in position than in *Wonambi* [2]. In posterior view, the posterolateral process is dorsally concave and forms a deep recess at the level of the basioccipital-otooccipital suture, similar to *Yurlunggur* [3].

**Parabasisphenoid**—The parabasisphenoid is roughly diamond-shaped in ventral view. The parasphenoid rostrum, including the cultriform process and cristae trabeculares, are not preserved with the exception of a thin spur of bone on the left side of the element. The position of the spur relative to the main body of the element implies a wide, parallel-sided parasphenoid rostrum. Anteriorly, the parabasispheneoid processes (= basitrabecular processes of [8]) are distinct and vertically oriented. They are proportionally smaller than in *Wonambi* and *Yurlunggur* [9,10], and similar in relative size to the condition in some boines and pythonines. The parabasisphenoid processes are ovoid in cross-section, with anteroposteriorly oriented long axes. A sagittal crest that is the origin of m. protractor pterygoidei extends from between the basipterygoid processes to the basioccipital articulation. The crest increases in height posteriorly. Laterally, the parabasisphenoid possess has wide triangular “wings” as in most other alethinophidians [4]. Wide posterior Vidian canal openings are present at the posterior margins of the wings. The posterior openings are approximately equal in size, and are contained completely with the parabasisphenoid. The anterior openings of the Vidian canal are not preserved. The canals do not open anteriorly at the base of the parabasisphenoid processes as in *Wonambi* and *Yurlunggur*. A shallow anteroposterior groove extends along the internal surface of the parabasisphenoid at the base of the right wing. The groove corresponds in size and shape to the intracranial anterior opening of the canal described for many taxa [11,12]. The visible articular surfaces for the prootic and parietal are wide and smooth.

**Palatine**—The right palatine is preserved in dorsal view, but its anterior and medial margins are not preserved. Posterolaterally, an elongate, vertically angled process abuts the posteromedial margin of the maxillary dorsal process. The process extends to near the top of the dorsal process and approaches the prefrontal. A palatine-prefrontal contact, which is present in *Xenopeltis*, cannot be confirmed in *Sanajeh*. The medial margin of the element is incomplete, but the preserved inflection suggests a vaulted choanal tract resembling those in basal snakes and varanids. The maxillopalatine foramen is not present. The posterior margin is broken, and there is no preserved morphology to indicate the nature of the pterygoid articulation.

**Dentary**—The right dentary is preserved in lateral view and a broken portion of the left dentary is preserved in dorsal view. The element is gracile and elongate with straight dorsal and ventral margins, as in *Xenopeltis*, *Loxocemus*, *Wonambi*, *Yurlunggur*, and derived macrostomatans. A single large mental foramen is presented on the anterolateral margin. Four widely spaced teeth are preserved in the right dentary, extending from the anterior margin of the element to the elongate postdentary process

**Dentition**—Dentary teeth are large, broad, and posteriorly angled, but not recurved as in derived alethinophidians. Preserved enamel surfaces are smooth without any evidence of plicidentine at the crown bases.

**Precloacal Axial Skeleton**—Only elements from the precloacal region of the axial skeleton are preserved. Vertebrae are preserved primarily in dorsal view, and several elements are transversely sheared. The best-preserved elements are a series of four articulated precloacal vertebrae in dorsal view, just ventral to the right dentary (Figure S9). Anteriorly, the zygosphene is prominent with a dorsal margin that is straight to gently concave with a slight indentation between the widely spaced articular facets. The anterior margin of the zygosphene is approximately straight, as in most taxa and variably in *Wonambi* [4]. The zygosphenal articular facets are ovoid and dorsolaterally angled, resembling most alethinophidians. Prezygapophyses are not readily visible, but the few partially preserved specimens do not possess well-defined accessory processes. The shape of the interzygapophyseal ridge ranges from angulated in more anterior elements to shallowly concave in elements from the middle and more posterior regions of the precloacal skeleton.

The neural spine is anteroposteriorly elongate, extending from the base of the zygosphene to overhang the neural arch. In lateral view, the spine is lower than in most alethinophidians, with strongly posteriorly slanted anterior and posterior margins, and a long, horizontal dorsal margin. The neural arch is vaulted, with an elevated posterior margin that approaches the dorsal margin of the neural spine in lateral view. In dorsal view, the posteromedian notch of the neural arch is well developed and has shallowly convex margins. The posterior margin of the neural spine originates on the posterior surface of the neural arch at the apex of the posteromedian notch. Small parazygantral foramina are present within shallow fossae on either side of the zygantrum on the posterior margin of the neural arch. The foramina are similar in size and configuration to those of medium-sized taxa such as *Najash*, *Patagoniophis*, *Alamitophis*, but not large-bodied taxa such as *Madtsoia*, *Wonambi*, and *Gigantophis* [13–16].

Synapophyses are preserved primarily in dorsal view. The processes are weakly divided into separate parapophyseal and diapophyseal facets, and extend laterally beyond the prezygapophyses, as in *Madtsoia* *bai* [14]. Transversely sheared elements preserve the margins of the cotyle and condyle, indicating that the articular surfaces were approximately circular as in alethinophidians and some scolecophidians, but not in outgroup squamates.

**Literature Cited in TEXT S4**

Lee MSY, Scanlon JD (2002) Snake phylogeny based on osteology, soft anatomy and ecology. Biol Rev 77: 333–401.

Scanlon JD (1997) *Nanowana* gen. nov., small madtsoiid snakes from the Miocene of Riversleigh: sympatric species with divergently specialized dentition. Mem Queensl Mus 41: 392–412.

Scanlon JD (2006) Skull of the large non-macrostomatan snake *Yurlunggur* from the Australian Oligo-Miocene. Nature 439: 839–841.

Rieppel O, Kluge AG, Zaher H (2002) Testing the phylogenetic relationships of the Pleistocene snake *Wonambi naracoortensis* Smith. J Vertebr Paleontol 23: 812–829.

Frazzetta TH (1999) Adaptations and significance of the cranial feeding apparatus of the sunbeam snake (*Xenopeltis unicolor*). J Morphol 239: 27–43.

Rieppel O (1977) Studies on the skull of the Henophidia (Reptilia: Serpentes). J Zool London 181: 145–173.

Apesteguía S, Zaher H (2006) A Cretaceous terrestrial snake with robust hindlimbs and a sacrum. Nature 440: 1037–1040.

Kluge AG (1991) Boine snake phylogeny and research cycles. Misc Publ Mus Zool Univ Michigan 178: 1–58.

Scanlon JD (2003) the basicranial morphology of madtsoiid snakes (Squamata, Ophidia) and the earliest Alethinophidia (Serpentes). J Vertebr Paleontol 23: 971–976.

Scanlon, JD (2005) Cranial morphology of the Plio-Pleistocene giant madtsoiid snake *Wonambi naracoortensis*. Acta Palaeontol Polonica 50: 139–180.

Underwood G (1967) A contribution to the classification of snakes. London: Trustees of the British Museum (Natural History). 179 p.

Maisano JA, Rieppel O (2007) The skull of the Round Island boa, *Casarea dussumieri* Schlegel, based on high-resolution X-Ray Computed Tomography. J Morphol 268: 371–384.

Albino A (2000) New record of snakes from the Cretaceous of Patagonia (Argentina). Geodiversitas 22: 247–253.

Simpson GG (1933) A new fossil snake from the *Notostylops* beds of Patagonia. Bull Am Mus Nat Hist 67: 1–22.

Hoffstetter R (1961) Nouveaux restes d'un serpent boïdé (*Madtsoia madagascariensis*) dans le Crétacé Supérieur de Madagascar. Bull Mus Nat d'Histoire Naturelle, 2e Sér 33: 152–160.

Smith MJ (1976) Small fossil vertebrates from Victoria Cave, Naracoorte, South Australia. IV. Reptiles. Trans Roy Soc South Australia 100: 39–51.
