# Supplementary material for: Predation upon Hatchling Dinosaurs by a New Snake from the Late Cretaceous of India
Source: PLoS Biol. 2010 Mar 2;8(3):e1000322. doi: 10.1371/journal.pbio.1000322 (PMC2830453; doi:10.1371/journal.pbio.1000322)
Supplement: Text S5 — Ootaxonomic affinities of eggs at Dholi Dungri. (0.03 MB DOC) [file pbio.1000322.s017.doc]

**Text S5. Ootaxonomic affinities of eggs at Dholi dungri**

Thousands of eggs and egg fragments have been recovered from the Lameta Formation in central and western India. A diversity of egg types, or ootaxa, have been developed in order to differentiate these, as recently reviewed [1,2]. Here, our interest is to confirm that the crushed and uncrushed eggs belong to the same ootaxon. Comparisons of surface structure and microstructure (Figure S10) of eggshell chips taken from one of the uncrushed eggs (GSI/GC/2905) and from the crushed egg on the underside of the Gandhinagar block (GSI/GC/2906) suggest that they both pertain to the oogenus *Megaloolithus*. The sauropod eggs associated with the Dholi Dungri snake and sauropod hatchling have been described as *Megaloolithus dhoridungriensis* [3]. These eggs can be diagnosed as *Megaloolithus* with spherical eggs; size range 140–180 mm diameter; thickness 2.26–2.36 mm; shell units discrete; tall and conical units with height/width = 2.74. The eggshell comprises long, broad, and elongated conical shell units, which are broad in the upper zone and taper into the basal cap zone. The basal cap zone is about 1/6 the shell unit in thickness. Growth lines are strongly arched in the lower part but become more shallow upwards. *M. dhoridungriensis* pore canals are broad and visible throughout the thickness of the eggshell. Their size and density resembles those of *Megaloolithus* eggs from Pinyes, Spain, which are significantly more porous than titanosaur eggs from Auca Mahuevo, Argentina [4]. It is likely that the high-porosity *M. dhoridungriensis* eggs, like those from Pinyes, were covered by a layer of vegetation or sediment.

We calculated the volume of the *M. dhoridungriensis* egg preserved adjacent to the snake and sauropod hatchling (GSI/GC/2905) to be 2,145 cm^3^, based on a radius of 8 cm for a subspherical egg. Assuming a density of 1.08 g/cm^3^ [4], we calculate egg mass to be 2,316 g. Interestingly, whereas *M. dhoridungriensis* egg volume and mass are closer to those of Auca Mahuevo eggs than to Pinyes eggs, clutch size overlaps that of Pinyes eggs (Table 1). Thus, *M. dhoridungriensis* has intermediate mass and clutch size but clutch masses that are approximately half the mass of clutches of smaller, more numerous eggs from Auca Mahuevo or the larger, less numerous eggs from Pinyes. The implications of this apparent difference in clutch mass amongst probable titanosaur sauropods is not yet resolved, but it may suggest that there were differences in body size of the reproductively-mature females, differences in the numbers of clutches deposited per year [5], or differences in reproductive effort.

**localityembryo/hatchling?egg volume (cm^3^)egg mass (g)clutch sizeclutch mass (g)reference**Auca Mahuevo, Argentinayes1,5961,724~2543,099[4]Pinyes, Spainno4,8495,237947,132[4]Dholi Dungri, Indiayes2,1452,3166–1213,897–27,795[3]

Table 1. Egg and clutch sizes for *Megaloolithus* eggs from Argentina, Spain, and India. We recalculated egg volume for Auca Mahuevo and Pinyes eggs assuming spherical shape (volume = 4/3π x r^3^; or 0.524 x d^3^). This coefficient differs from the slightly smaller value of 0.51 used by [4], and as a consequence, the volumes reported here are slightly larger.

**Literature Cited** **in TEXT S5**

Khosla A, Sahni A (1995) Parataxonomic classification of Late Cretaceous dinosaur eggshells from India. J Palaeontol Soc India 40: 87–102.

Mohabey DM (2001) Indian dinosaur eggs: a review. J Geol Soc India 58: 479–508.

Mohabey DM (1998) Systematics of Indian Upper Cretaceous dinosaur and chelonian eggshells. J Vertebr Paleontol 18: 348–362.

Jackson FD, Varricchio DJ, Jackson RA, Vila B, Chiappe L (2008) Comparison of water vapor conductance in a titanosaur egg from the Upper Cretaceous of Argentina and a *Megaloolithus siruguei* egg from Spain. Paleobiol 34: 229–246.

Sander PM, Peitz C, Jackson FD, Chiappe LM (2008) Upper Cretaceous titanosaur nesting sites and their implications for sauropod dinosaur reproductive biology. Palaeontographica Abt A 267: 119–168.
