# Supplementary material for: Predation upon Hatchling Dinosaurs by a New Snake from the Late Cretaceous of India
Source: PLoS Biol. 2010 Mar 2;8(3):e1000322. doi: 10.1371/journal.pbio.1000322 (PMC2830453; doi:10.1371/journal.pbio.1000322)
Supplement: Text S6 — Phylogenetic analysis. (0.15 MB DOC) [file pbio.1000322.s018.doc]

**TEXT S6. PHYLOGENETIC ANALYSIS**

The majority of characters used in this analysis come from evaluation of the two most recent comprehensive morphological analyses of snake phylogeny [1,2]. Original references for character discovery are cited in those studies. Modifications and new characters are based on specimen observations generated in collection research by JJH (see Examined Specimens, below). Published descriptions were used to augment observations and character scorings for *Dinilysia* [3–6], *Najash* [2,7], Bolyeriidae [8–10], *Anomochilus* [11–14], and Amphisbaenia [15–20].

We have assumed the monophyly of several suprageneric taxa (e.g., Scolecophidia, Pachyophiidae) that have been corroborated in previous phylogenetic analyses and whose constituent taxa do not share potential synapomorphies with *Sanajeh*. Accordingly, we have excluded characters whose states vary only within those clades, as well as several characters that were found to be so polymorphic as to be not useful in character coding, or for which character observations could not be replicated. As a result, our character-taxon matrix is smaller than those of the analyses it is based on [1,2].

Unlike other analyses [2], we did not treat Anilioidea as a natural taxon *a priori*. Instead, we separated Anilioidea into its constituent major lineages, recognizing that morphological and molecular studies have not resolved monophyly of the clade [1,11,21]. Additionally, morphology of the fossil taxa *Wonambi* and *Yurlunggur* has been favorably compared to anilioids, and our analysis tests these potential homologies. Scorings for *Wonambi* and *Yurlunggur* are based on recent descriptions [1,22,23].

There is little unanimity among hypotheses of the relationships of snakes within squamates [24,25]. Consequently, we performed two separate phylogenetic analyses: one using Varanoidea (*Varanus*, *Heloderma*) as outgroup to snakes and the other using Amphisbaenia as outgroup to snakes [1].

We derived our phylogeny using a heuristic parsimony search in PAUP* 4.0b [26] using 10,000 random addition sequence replications. Decay indices were calculated using PAUP and MacClade [27].

**Character List**

We used characters published in the two most recent phylogenetic analyses of snakes. Characters most closely corresponding to those published in [2] are indicated by “AZ”, and those from [1] are indicated by “S”.

PREMAXILLA

1. Premaxillary teeth present (0), absent (1) [AZ4, S173].
2. Transverse process of premaxilla recurved (0) straight or concave (1) [AZ6, S1].
3. Ascending medial process of premaxilla elongate, approaching or contacting frontals (0), short, divides nasals only at anterior margin or not at all (1) [S2]. A long ascending process is present in *Anilius* and *Cylindrophis maculatus* among ingroup taxa.
4. Premaxilla-maxilla contact sutural or rigid articulation (0), flexible contact (1), separated (2) [S12]. *Wonambi* and *Yurlunggur* are scored as state 1 based on morphological similarities of the anteromedial maxilla with alethinophidians such as *Anilius* and *Xenopeltis*.

MAXILLA

1. Anteromedial maxillary shelf present (0), absent (1) [AZ40, S15].
2. Ascending process of maxilla present (0) absent (1) [AZ38, S14]. We regard the condition in *Loxocemus*, where the ascending process is the same relative height as in *Xenopeltis* and *Wonambi* [28] as primitive.
3. Palatine process of maxilla anterior to or at anterior margin of orbit (0), ventral to midline of orbit (1) [AZ42, S18].
4. Palatine process of maxilla pierced by large foramen (0), not pierced (1) [AZ43, S21].
5. Posterior margin of maxilla terminates at posterior margin of orbit (0), extends moderately beyond orbit (1), extends far beyond orbit- orbit at midlength or toward anterior end of maxilla (2) [AZ41, S23].

NASAL

1. Anterior margin of nasals restricted to posteromedial margins of nares (0), extends anteriorly toward tip of rostrum (1) [S24]. The condition is variable in amphisbaenians [16].
2. Anterodorsal surface of nasal narrow (0), broad (1) [AZ7, S25]. Character states rescored following [1] based on observation of *Heloderma* and *Varanus*.
3. Medial flanges of nasal articulate with median frontal pillars (0), flanges do not articulate with median frontal pillars (1) [AZ8]. Scored as inapplicable for taxa lacking median frontal pillars.
4. Lateral flanges of nasal articulate with anterior margin of frontals (0), are separated from frontals (1) [S27].
5. Posterolateral margin of nasal contacts posteromedian margin of prefrontal (0), elements in contact along most of their length (1), contact between elements with interfingering of nasal and prefrontal margins (2) nasals do not contract prefrontals (3) [S33].

PREFRONTAL

1. Discrete prefrontal apex [29] absent (0), present (1). A triangular anterior apex of the prefrontal that does not articulate with the dorsal surface of the maxilla is present in erycines, ungaliophiines, boines, pythonines, and weakly in tropidophiines among examined taxa.
2. Lacrimal present (0), absent (1). The lacrimal is absent in all extant snakes and fossils referred to Serpentes where the morphology of the anterior orbit is preserved. Maxillary-prefrontal articulation in *Sanajeh* indicates the absence of a distinct lacrimal. The condition in amphisbaenians is based on the interpretation of [16].
3. Prefrontal-maxillary articulation continuous along length of prefrontal (0), “peg and socket” articulation [11] (1), restricted to posterior portion of prefrontal (2) [AZ22, S34]. The “peg and socket” condition describes the morphology in anilioids, where a dorsal extension of the maxillary dorsal process is received in an excavation of the lateral margin of the prefrontal.
4. Ventrolateral process of prefrontal absent (0) contacts maxilla (1), contacts maxilla and palatine (2), contacts palatine (3). [AZ19, S36]. A thin ventrolateral process separates the external nares from the orbit in *Leptotyphlops* species among scolecophidians. Pachyophiids are scored based on *Eupodophis* [30].
5. Ventromedial process of prefrontal absent (0), present, low (1), present, high (2) [AZ20].
6. Lacrimal foramen located between prefrontal and maxilla (0), completely enclosed with prefrontal (1) [AZ18, S45]. This character is polymorphic within Amphisbaenia and Boinae. Scoring for *Loxocemus* differs from [1], based on specimen observation.

FRONTAL

1. Median frontal pillars at anterior margin of frontals absent (0), present, incomplete (1), present, complete (2) [AZ24, S56]. The median frontal pillars do not completely separate the olfactory nerve tracts in *Wonambi*, *Yurlunggur*, and uropeltines.
2. Frontal-prefrontal articulation occurs at anterolateral margin of frontal (0), prefrontal articulates along lateral embayment of frontal (1) [S41].
3. Frontal-parietal suture approximately transverse (0), strongly concave anteriorly (1) [AZ33, S54]. The suture in amphisbaenians is apomorphically transformed relative to the conditions coded here.

POSTORBITAL

1. Dorsal process of postorbital simple or broadly concave at frontoparietal suture (0), sharply forked at articulation with parietal (1) [AZ29, S48]. Scoring follows [2] based on observation of *Heloderma* and *Varanus*.
2. Posterior margin of orbit formed primarily by dorsal extension of jugal (0), formed primarily by ventral extension of postorbital (1), postorbital absent- posterior margin open (2) [AZ27, S51]. The posterior margin of the orbit is open in extant amphisbaenians; however, the jugal forms the posterior margin of the orbit in fossil taxa [16].
3. Jugal present (0), absent (1) [S46]. We consider identification of the apomorphically broad, triangular element identified by [1] as a jugal in *Yurlunggur* a tenuous hypothesis, but score the taxon following that description. The jugal is present in fossil amphisbaenians and *Rhineura floridana* [16,17].

PARIETAL

1. Anterior margin of parietal laterally expanded to form portion of postorbital process (0), anterior margin not expanded (1) [AZ30, S50]. Expansion of the anterolateral margin of the parietal to contribute to the postorbital process is absent in anilioids and species of *Leptotyphlops* among scolecophidians. The primitive state was observed in *Loxocemus* contra [1]. *Xenopeltis* possesses a distinct lateral process although the postorbital is absent. Character states rescored from [2] based on observation of *Heloderma* and *Varanus*.
2. Lateral margins of the braincase open anterior to prootic (0), descending lateral processes of parietal enclose braincase (1).
3. Lateral margins of parietal approximately smooth (0), lateral margins include transverse crest (1) [A31, S67].
4. Parietal participation in posterior border of optic foramen absent or shallow (0), well-developed (1), optic foramen located within frontal (2) [AZ34, S61]. Character state coding follows [2]; the apomorphic condition in scolecophidians is scored following [1].
5. Parietal participates in anterior margin of trigeminal foramen (0), excluded from foramen by prootic (1) [AZ77, S132].
6. Sagittal crest absent (0), weakly developed, restricted to posterior margin of parietal (1) strongly developed (2) [AZ32].
7. Supratemporal margin of parietal laterally expanded (0), not expanded (1) [AZ35, S66]. Character delineation follows [2]. Scoring for *Wonambi* based on [1].

SUPRATEMPORAL

1. Supratemporal present (0), absent (1) [AZ 47, S69 part].
2. Distal end of supratemporal confluent with otooccipital (0), distal end is a free-ending process forming quadrate suspensorium (1) [AZ46].
3. Anterior margin of supratemporal posterior to or above posterior border of trigeminal foramen (0), anterior to posterior border (1) [AZ 44, S72].
4. Supratemporal L or C -shaped (0), short and broad (1), elongate, strap-shaped (2). Character scoring for *Wonambi* and *Yurlunggur* is based on the shape of the supratemporal articular surfaces of the prootic-otooccipital; scoring for *Najash* is based on [7].
5. Supratemporal articular surface of prootic-otooccipital smooth or recessed relative to parietal margin (0), rugose (1) [AZ45].
6. Quadrate suspensorium approximately at level of occipital condyle (0), anterior to condyle (1), extended posteriorly beyond condyle (2). Similar to S70, but character state coding is reversed based on observation of *Heloderma* and *Varanus*.

QUADRATE

1. Suprastapedial process present (0), present, process longer than quadrate shaft (1), absent (2). [AZ66, S74]. We infer a prominent process in *Sanajeh* based on the long articular facet along the ventrolateral margin of the supratemporal. Uropeltines and *Anomochilus* possess suprastapedial processes that exceed the length of the vertical shaft of the quadrate.
2. Long axis of quadrate oriented anteroventrally (0), approximately vertical (1), oriented posteroventrally (2), strongly anteriorly angled, approaching horizontal (3). [AZ65, S78].
3. Stylohyal process not fused to quadrate shaft (0), fused to shaft (1) [AZ67, S73]. Amphisbaenians are scored as inapplicable for this character due to the highly derived orientation and function of their extracolumella [16].

PROOTIC

1. Laterosphenoid absent (0), present, divides trigeminal foramen (1) [AZ78, S130]. We score *Yurlunggur* following [1] and *Wonambi* is scored as unknown after [23].
2. Hyomandibular branch of facial nerve exits skull outside the opening of the trigeminal foramen (0), exits inside the opening of the trigeminal foramen (1) [AZ81, S133].
3. Juxtastapedial recess absent (0), present (1) [AZ71]. This character is modified from [2] based on reversal of character state order in our character 48. The condition described as a posteriorly open juxtastapedial recess is a derived character in our analysis, based on the reduction of crista tuberalis. *Dinilysia* is scored as lacking a juxtastapedial recess based on the occurrence of the stapedial footplate and lateral aperture of recessus scala tympami on the lateral surface of the skull and the absence of a crista circumfenestralis [5]. Similarly, *Najash* is scored as primitive, as it appears to possess only a slight expansion of the anteroventral margin of the prootic-stapedial contact, prohibiting occurrence of a pericapsular recess lateral to the footplate.
4. Prootic exposed in dorsal view medial to supratemporal (0), not exposed- supratemporal contacts supraoccipital (1) [AZ80, S136]. The dorsal margin of the prootic is concealed by the wide parietal in *Heloderma*.

Otooccipital

1. Crista tuberalis well developed, obscuring jugular foramen in lateral view (0) greatly reduced, exposing jugular foramen (1) [AZ74]. Character state coding is reversed from [2], based on the condition observed in *Varanus*. All occipital cristae are reduced in *Heloderma*, a condition that appear to be apomorphic in comparison with *Estesia* [31] and *Varanus*.
2. Accessory process [32] of the crista interfenstralis does not contribute to the crista circumfenestralis (0), extends laterally to form distinct part of ventral margin of the crista circumfenestralis (1) [AZ73].
3. Otooccipitals broadly separated by supraoccipital (0), meet to form dorsal margin of foramen magnum (1) [AZ75, S141].
4. Paraoccipital extension overhangs foramen ovalis (0) extension greatly reduced or absent (1) [AZ70, S137]. The paraoccipital portion of the otooccipital is not preserved in *Najash* [7]; however, the lateral extension of the parietal and prootic above the foramen ovalis [7, fig. 3] indicates that the paraoccipital extension was present.

STAPES

1. Stapedial shaft straight (0), strongly angulated (1) [AZ68, S144]. Inferred for *Najash* following [7].
2. Stapedial shaft elongate and slender (0), shaft short and robust (1) [AZ69, S145].
3. Stapedial footplate small (0), large, dominates posterolateral surface of otic capsule (1). A greatly enlarged stapedial footplate is present in *Najash*, *Dinilysia*, and *Xenopeltis*. Character delineation is based on comparison of the stapedial footplate with the otic capsule, regardless of development of the crista circumenfenestralis.

BASIOCCIPITAL

1. Elongate sagittal crest for origin of m. protractor pterygoidei does not extend onto basioccipital (0), extends onto basioccipital (1). Weakly developed crest occur in multiple taxa, however a tall crest extending ventrally beyond the level of the basioccipital-parabasisphenoid suture occurs only in *Sanajeh*, *Wonambi*, *Yurlunggur*, boines, pythonines, and some colubroids.
2. Anterolateral crests of basioccipital absent (0), present (1) [AZ87]. This character was considered a synapomorphy of *Wonambi* + Boinae by [2], but crests are also present in pythonids, and variably in *Loxocemus*, *Xenopeltis*, and colubroid taxa.
3. Basioccipital-parabasisphenoid suture between fenestra ovalis and trigeminal foramen (0), at level of fenestra ovalis (1), at level of trigeminal foramen (2) [S121, AZ91].
4. Basioccipital posterolateral processes short and narrow, do not extend toward posterior margin of occipital condyle, (0), wider than condyle and long, combine with crista tuberalis to extend to approximate posterior margin of occipital condyle (1). The crista tuberalis extends posterolaterally to contact a wide, posteriorly extensive lateral projection of the basioccipital in *Wonambi*, *Yurlunggur*, and *Sanajeh*. Smaller crests are present in other ingroup and outgroup taxa (e.g., *Anomochilus*) [9]. See also [33], character 122.
5. Basioccipital contributes to ventral margin of foramen magnum (0), basioccipital excluded by medial contact of otooccipitals (1) [S142]. Scoring for amphisbaenians is based in part on character 106 in [16].

PARABASISPHENOID

1. Cultriform process of parabasisphenoid does not extend anteriorly to approach posterior margin of choanae (0), approaches posterior margin of vomera (1).
2. Parasphenoid rostrum no wider than width of anterior margin of crista trabeculares (0), elongate, wider than width of crista trabeculares (1), broad and triangular (2) [AZ92, S116]. The anterior basisphenoid region between the level of the basipterygoid or parabasisphenoid processes (when present) and the parasphenoid cultriform process is approximately the same width as the parasphenoid process in *Heloderma*, *Varanus*, and some pythonids and boids. *Wonambi*, *Yurlunggur*, *Loxocemus*, *Xenopeltis, Cylindrophis, Anilius*, bolyeriids, *Acrochordus*, and colubroid taxa possess a rostrum that is narrower than the body of the element at the level of pterygoid contact, but broader than the parasphenoid process. The rostrum is broadly triangular in scolecophidians, *Anomochilus*, and uropeltines. All three conditions are present in amphisbaenians [17].
3. Parabasisphenoid rostroventral surface flat or broadly convex (0), concave (1) [S116]. The ventral surface of the parabasisphenoid is concave in amphisbaenians, scolecophidians, *Yurlunggur*, *Wonambi*, anilioids, and multiple colubroids,
4. Crista trabeculares absent or poorly developed (0), elongate and prominent (1) [AZ89].
5. Basipterygoid processes present (0), absent (1) [AZ88, S117]. Different embryological origins of the basipterygoid processes (basal plate) of non-snake squamates and the ventral parabasisphenoid projections and wings (dermal parasphenoid, trabeculae) in extant snakes suggest that the two conditions are not homologous [34,35]. We score all snakes that have either smooth ventral basisphenoid margins, (or possess ventral projections in conjunction with a platybasic skull, see below) as having lost the basipterygoid process. Discrimination between the condition in snakes and other squamates has been corroborated in multiple studies [34].
6. Parabasisphenoid (=basitrabecular) processes absent (0), present (1). Distinct ventral projections [34] are present in boids, pythonids, and erycines among extant taxa. Character scoring for *Najash*, *Dinilysia*, *Sanajeh*, *Wonambi*, and *Yurlunggur* is based on the inference of a platybasic skull [35,36] (including parasphenoid contribution to the ventral projections and lateral wings) from the size and length of the parabasisphenoid. Contra [2], we observed distinct processes only in *Cylindrophis maculatus* among anilioid taxa.
7. Parabasisphenoid wings absent (0), wings present (1) [AZ85, S119]. Ascending margins of wings appear weakly developed *Wonambi* and *Yurlunggur*, however we maintain character scoring of [1] for both taxa.
8. Anterior opening of the Vidian canal opens on external surface of the element at level of parabasisphenoid processes (when present) (0), primary anterior opening is on the internal surface of the parabasisphenoid lateral or anterolateral to sella turcica (intracranially) (1) [AZ82, S124]. We observed the primitive condition in specimens of *Loxocemus* as well as boine taxa.
9. Dorsum sellae of sella turcica well developed (0), poorly-developed (1) [AZ84, S128].
10. Elongate sagittal crest for origin of m. protractor pterygoidei absent (0), present (1) [S120].

PTERYGOID

1. Pterygoid teeth absent (0), present (1).
2. Ectopterygoid processes of pterygoid discrete, well-developed processes (0), processes greatly reduced or absent (1) [S101].

ECTOPTERYGOID

1. Ectopterygoid articulates with posteromedial surface of maxilla (0), articulates with dorsomedial surface of maxilla (1) [AZ62]. We infer a dorsally overlapping ectopterygoid in *Sanajeh* based on the wide, concave morphology of the posterior process of the maxilla.

PALATINE

1. Anterior process of palatine absent (0), present, dentigerous (1), present, flat, broad, toothless plate (2) [AZ48, S94].
2. Maxillopalatine foramen present (0), absent (1) [AZ55, S98].
3. Maxillary process situated anterior to the approximate midpoint of palatine (0), situated toward posterior margin of palatine (1) [S97].
4. Choanal process broad and vaulted over ductus nasopharingeous (0), forms narrow, curved process (1), small, does not contact vomer (2) [AZ49, S91, 92].
5. Choanal process without expanded anterior flange articulating with vomer (0), with anterior flange (1) [S93]. Character scoring for *Dinilysia* differs from [1] based on [3].
6. Palatine-pterygoid articulation interdigitate (0) mediolateral clasping projections of palatine receive anterior pterygoid process (1), elongate projection includes long medial process of palatine (2), simple overlap (3) [AZ50, S99].
7. Palatine contacts ectopterygoid (0), does not contact ectopterygoid (1).

VOMER

1. Vomer excluded from the lateral margin of opening for Jacobson’s organ by premaxilla and/or maxilla (0), septomaxilla (1), forms the posterolateral margin of the opening (2) [AZ13, S84].
2. Vomeronasal nerve does not pass through vomer (0), enters through large foramen (1), enters through cluster of foramina (2) [AZ14, S85].
3. Vomerine posterior horizontal processes elongate (0), short (1) [AZ15, S88]. Character state scoring follows [2], based on observations of *Heloderma* and *Varanus*. Condition in pachyophiids is scored for *Eupodophis* [30].
4. Vomerine posterior vertical processes reduced or absent (0), well developed (1) [AZ16, S89]. Character state scoring follows [2], based on observation of *Heloderma* and *Varanus*.

SEPTOMAXILLA

1. Median vomeronasal fenestra formed by septomaxilla and vomer (0), ventral margin of septomaxilla contacts dorsal margin of vomer along its length (1) [AZ12, S86].
2. Dorsolateral process [34] absent (0), present (1) [AZ9, S80]. Character states are simplified from [1,2], because the refined multistate coding of those studies could not be accurately reproduced in examined specimens**.**
3. Septomaxilla does not articulate with median frontal pillars (0), articulates with pillars (1) [AZ10, S82]. Scored as inapplicable for taxa lacking median frontal pillars.

DENTARY

1. Anteromedial margin of dentaries possess symphyseal articular facet (0), dentaries lack symphyseal articular facet (1). *Najash* is the only snake to possess a bony mandibular symphysis [7].
2. Mental foramen multiple (0), singular (1) [AZ 95, S148].
3. Posterior dentigerous process of dentary absent (0), present, short (1), present, elongate (2) [AZ 93, S149-150].

CORONOID

1. Coronoid present (0), absent (1) [AZ97, S160].
2. Coronoid forms entirely of coronoid process (0), coronoid forms anterior margin of coronoid process (1) [AZ99, S160, 164]. The coronoid is laterally overlapped by the dentary and compound bone in most amphisbaenian taxa, but it forms the entire process in medial view. In alethinophidian snakes, the coronoid is restricted to the anterior margin of the process in all views.
3. Coronoid posteroventral process present (0), absent (1) [AZ98, S161]. We score *Wonambi* and *Yurlunggur* as unknown contra [1] in the absence of a recovered coronoid for either taxon.

COMPOUND BONE

1. Discrete surangular and articular postdentary elements (0), fusion of surangular and articular into compound bone (1). A compound element is present in most amphisbaenians [16].
2. Medial margin of adductor fossa low with no crest (0), low crest present (1), distinct, tall crest present (2) [AZ94, S166]. Recoded to differentiate between the low crest of most snake taxa and the extremely tall crest in boids, pythonids, and some colubroids.

DENTITION

1. Teeth strongly posteriorly angled but straight (0), strongly recurved posteriorly (1) [AZ3].
2. Plicidentine present (0), absent (1) [AZ2, S172]. *Yurlunggur* and *Wonambi* are scored following [37].
3. Tooth implantation pleurodont (0), “modified pleurodont” [18] with interdental ridges forming alveoli (1) [AZ1].

AXIAL SKELETON

1. Well-developed, consistently distributed paracotylar foramina absent (0), present (1). Small, irregular foramina are present dorsolateral to the cotyle in some specimens of *Varanus* and *Heloderma*. Well-developed foramina lateral to the cotyle are present in derived macrostomatans, pachyophiids, *Wonambi*, and *Yurlunggur*.
2. Prezygapophyseal accessory processes absent (0), short (1), elongate (2) [AZ103, S200].
3. Synapophyses extend laterally beyond prezygapophyses (0), prezygapophyses extend laterally beyond synapophyses (1) [AZ113]. Scored for non-pachyostotic regions of the precloacal vertebral column in pachyophiids.
4. Cotyle shape of precloacal vertebrae strongly ovoid (0), approximately circular (1) [AZ108, S193].
5. Neural spine present (0), absent or greatly reduced without tall vertical lateral margins (1) [AZ106, S190]. We observed well-developed neural spines in *Loxocemus* contra [2].
6. Posterior median notch of neural arch absent (0), weakly developed (1), well-developed, exposing cotyle (2) [AZ107, S191].
7. Parazygantral foramina absent (0), present, small multiple foramina (1), present, large paired foramina (2). Large, paired foramina occur in *Najash*, *Sanajeh*, *Yurlunggur*, *Wonambi*, some erycines and boines, and colubroids.
8. Subcentral foramina absent (0), symmetrical and paired (1), asymmetrically sized or singular (2) [AZ104, S199]. Large, paired foramina are variably present along the ventrolateral margins of the centrum in examined amphisbaenians.
9. Ventral margin of centra smooth (0), median prominence extending from cotyle to condyle on ventral surface (1) [S202].
10. Precloacal vertebrae number less than 100 (0), more than 100 (1). Amphisbaenian vertebral counts range from 64 to 145 [38]. All extant and known fossil snakes possess more than 100 precloacal vertebrae.
11. Hypapophyses restricted to anterior most precloacal vertebrae (0), present throughout precloacal skeleton (1) [S201].
12. Subcentral paralymphatic fossae [39] on posterior precloacal vertebrae absent (0), present (1). Presence of fossae is indicated by deep excavation between the centrum and synapophyses of posterior precloacal vertebrae that house the enlarged perilymphatic system.
13. Cloacal lymphapophyses absent (0), present, three or fewer (1), present, four (2) [AZ111-112, S203]. Among pachyophiids, *Pachyrhachis* possesses three lymphapophyses *Eupodophis* lacks the processes, and *Haasiophis* possesses possibly 5 lymphapophyses.
14. Fused haemopophyses on caudal vertebrae absent (0), present (1) [AZ101, S206]. Weakly bifurcated haemopophyses are present on the ventral surface of caudal vertebrae in uropeltines among anilioids. Presence in *Najash* is based on [7].
15. Caudal vertebrae number greater than 50% of precloacal number (0), approximately 10% or less than precloacal number (1).
16. Tuber costae absent from ribs (0), tuber costae present (1) [S207]. Pachyophiids were previously considered to lack tuber costae based on observations of pachyostotic elements. Ribs anterior and posterior to pachyostotic segments possess tuber costae in *Pachyrhachis*, *Haasiophis*, and *Eupodophis*.

APPENDICULAR SKELETON

1. Pectoral girdle and forelimbs present (0), absent (1).
2. Tibia, fibula, and hind foot present (0), absent (1) [S212; listed as “48” in the original publication].
3. Femur with well-developed femoral trochatera present (0), femur short, lacking trochantera (1) femur absent (2) [AZ115, S212, listed as “48” in the original publication]. The polymorphic scoring for amphisbaenians is based on the presence of greatly reduced elements in *Bipes* and *Blanus* [15].
4. Pelvis braced against axial skeleton via sacral vertebrae (0), pelvis not braced against axial skeleton (1), pelvis absent (2) [A116, 119, S210, listed as “46” in the original publication]. Scoring for amphisbaenians follows [15].

**Character-Taxon Matrix**

Scorings for 116 characters in 23 operational taxonomic units are presented below. Scorings inside parentheses indicate polymorphisms. Unordered changes were assumed for all multistate characters. The text is formatted so that it can be copied and placed in PAUP.

#NEXUS

BEGIN DATA;

DIMENSIONS NTAX=23 NCHAR=116;

FORMAT SYMBOLS= " 0 1 2 3" MISSING=? GAP=- ;

MATRIX

Amphisbaenia

00{01}000--{01}{01}{01}-03010{01}{01}{01}0{01}--{02}{01}01{01}-{01}{12}01----123-0-0-1-0011000{01}0{01}1{012}10{01}000100000100000010000-000000{01}{01}0000110100{01}0{01}00{01}111{01}1{12}1

Varanoidea

000000--000-0{03}00000-000000000--000000-00000-000-00000{01}0{01}000000000000{01}000000000000000-000000000000000000{02}000000000000

Najash rionegrina

???????????????????????????10?00000010?01000001?001?1?00???110110??0?????????????????00??????0?0?0011121110?10?1?000

Dinilysia patagonica

???1000?111-00010210010010011002000010001000001010111000001110110??01000000?11???????1?10001001100110101110?????1???

Scolecophidia

1{01}11001-011-0101-{01}0-000-2{01}{01}102001{01}0---1230001-101?11000{012}00121010{01}11001-010103-101100010000010010021{01}100{12}{01}101101111{12}1

Anilius scytale

00010000111000011111201-21110001000010101010101010110001001111101110100110002111000101100101101101111101110110111111

Anomochilus

10110000011001011110201-2111000010001011001010101011000101121010111000-2000021111101011101110011?????????1????1?1111

Cylindrophis

10{01}1{01}00011100001111020101111000100001010101{01}1010{01}01100010111101{01}1110100100002111111101110101101101111101110110111111

Uropeltinae

101{01}0000110001011110101-21110{02}0{01}{01}1----1100101-101{01}1100010{01}1110101110000200002111111101100111101101111101110111111122

Sanajeh indicus

???1000?2??????102????1??1?10?0200101000??00110110??01121???0?111??1??1?1?0??1???????112???1?011?001012?110????1????

Wonambi

???110002???0??10???110???01100210?121?????0110110??011211?1111100111?11100111???????1120??11111100102{12}1110?20?1????

Yurlunggur

10110000210?030101?0111100011002?0?021?010001101?0??0112111111??001110111001111110?1?1020??1?11110{01}102211101?0?1????

Pachyophiidae

1111100?{02}10?000101??2{01}0011010??2101?2?0211???1??0100?0??0?11??11????10{01}1?11??1??0????11200011111101102011101{012}{01}{01}11011

Xenopeltis

001100012110020103102010210100011010100010101111111110{01}200110110111111111010111101110112011111110111120{12}110121011122

Loxocemus bicolor

001200012110020101102000110100021010200211111111110000{01}200110110101110111010{12}111111101120111111101110201110121011111

Erycinae

1112111011000{01}1121202000{12}10101{01}{12}10112002111111011100000200110111111{01}101{01}11{01}021111111011201112111011102{012}1110121011111

Ungaliophiinae

1112111101000011212020001101010110102{01}021111110111000002001101101110101111102111111101121--1111101110201110121011111

Boinae

1112111021000{01}11212{01}2001110101{01}210112122{12}1111101{01}1000112001001111{01}11101111{12}0{12}1111111011201112111{01}11102{12}1110121011111

Pythonidae

011211102100001121212001110101{01}210112122{12}11{01}1101110001120{01}10011111111{01}11011021110111011201112111011102{01}1110121011111

Tropidopheinae

1112110001001311211020001101011110102{01}0211111100110000000011011011101011{01}0001111{01}11101121--11111111102011111210111{12}{12}

Boyleriidae

11121101210013012110200011010101101020001111110111000002001101101111101110102111011101120111211111110201110121011122

Acrochordus

11121101210113012211200011011111101121222111110011000002001101101111111110001122101111021--11111111102011111210{01}1122

Colubroidea

111{12}1{01}11{012}10{01}13012{02}{01}120001101011{12}101{01}2{01}{02}2{12}111110111000{01}{01}20011{01}110111{01}1111{01}0101122101111121--121111{12}1102{012}1111121011122

;

END;

BEGIN ASSUMPTIONS;

OPTIONS DEFTYPE=unord PolyTcount=MINSTEPS;

END;

**Trees**

Two heuristic analyses of the character-taxon matrix employing different outgroup strategies were performed. In one analysis trees were rooted with Varanoidea, in the other trees were rooted with Amphisbaenia. Outgroup choice had minimal effects on the interrelationships of the ingroup. Both analyses produced a single most parsimonious tree in which *Sanajeh* is resolved as a non-macrostomatan alethinophidian (see Figure S11 for topology and tree metrics). The topologies of the two trees are identical except for the position of *Najash* relative to Scolecophidia and *Dinilysia*. When trees are rooted with Varanoidea, *Najash* is resolved as the basalmost snake; when trees are rooted with Amphisbaenia, *Najash* is resolved as sister-taxon to *Dinilysia*. Rooting with Amphisbaenia produced a shorter tree that has stronger support values at basal nodes that tress rooted with Varanoidea. The monophyly of and interrelationships within Alethinophidia are robustly supported using either outgroup. Substantial character evidence nests Pachyophiidae within Alethinophidia as the basalmost macrostomatan and supports the monophyly of *Sanajeh* and the Australian ‘madtsoiids’ (*Wonambi* and *Yurlunggur*).

To further evaluate the strength of our phylogenetic hypothesis, we ran constrained analyses designed to determine the parsimony debt incurred by two competing hypotheses, given our data matrix (Figure S12). As with the initial analyses, we employed two different rooting strategies, but topological results and parsimony debts were nearly identical under either outgroup hypothesis. The first constraint analysis fixed *Wonambi*, *Yurlunggur*, *Dinilysia*, and Pachyophiidae as basal snakes with unconstrained relationship to one another, following the results of [1]. This first constraint analysis resulted in two and five equally parsimonious trees 21 and 24 steps longer than unconstrained results rooted with Varanoidea and Amphisbaenia, respectively. The second constraint analysis specified a sister-taxon relationship between *Wonambi* and Boinae, following the results of [2], and resulted in a single tree that was 21 steps longer than the unconstrained results. Trees constrained to include a *Wonambi*-Boinae sister-group relationship recovered nearly the same topology as the original, with *Sanajeh* and *Yurlunggur* basal to macrostomatans within crown-group snakes. Trees constrained to resolve *Wonambi*, *Yurlunggur*, Pachyophiidae, and *Dinilysia* in a basal position resolved *Sanajeh* outside of crown-group snakes.

**Examined Specimens**

Institutional abbreviations: BMNH, the Natural History Museum, London; HUJ-PAL, Hebrew University, Jerusalem Palaeontological Collection; MVZ, University of California Museum of Vertebrate Zoology; ROMV R Royal Ontario Museum Paleobiology Recent Collection; TMM M, Texas Memorial Museum Modern; TMMVP, Texas Memorial Museum Vertebrate Paleontology Laboratory; UCMP, University of California Museum of Paleontology; UMMZ, University of Michigan Museum of Zoology; USNM, United States National Museum, Smithsonian Institution. An asterisk (*) indicates use in estimating body length of *Sanajeh indicus*.

Varanoidea: *Heloderma*: *H. horridum*, ROMV R-278; *H. suspecturm* ROMV R-279, ROMV R-556. *Varanus*: *V. komodoensis* ROMV R-7565, *V. flavescans* ROMV R-758, *V. niloticus* ROMV R-7303; *V. salvator* ROMV R-136.

Amphisbaenia: *Amphisbaena fenestrata*, MVZ 204284; *A. gonavensis*, MVZ 191810, MVZ 191813; *Anops kingii*, MVZ 200486; *Bipes biporus* UCMP 1198020, UCMP 137630, UCMP 137868; *Cynisca leucura*, MVZ 75398.

Scolecophidia: *Leptoyphlops dulcis* USNM 161288*; *Leptotyphlops* *humilis* USNM 222794; *Leptotyphlops* *albifrons* USNM 563525; *Ramphotyphlops* *braminus* USNM 512273, USNM 539348;*Typhlops punctatus* BMNH 1911.6.9.2*, *Typhlops reginae* ROMV R-169.

*Anilius scytale* BMNH 56.10.16, BMNH 58.8.23.48; TMM M 8281; USNM 204080.

*Cylindrophis*: *C. maculatus* BMNH 1930.5.8.50; *C. ruffus* BMNH 1930.5.8.47; UCMP 136995; USNM 523565*.

Uropeltinae: *Brachyophidium rhodogaster* TMMVPL M-8283; *Melanophidium* *punctatum* BMNH 1930.5.8.119; *Platyplecturus madurensis* BMNH 1930.5.8.114; *Plecturus perroti* BMNH 1930.5.8.106; *Uropeltis ceylcanicus* BMNH 1930.5.8.70-71, 1930.5.8.85-88, BMNH 1930.5.8.91, BMNH 1964.967; *Uropeltis woodmasoni* TMMVPL M-8284.

Pachyophiidae: *Haasiophis terrasanctus* HUJ-PAL EJ 695; *Pachyrhachis* *problematicus* HUJ-PAL 3659, HUJ-PAL 3775.

*Xenopeltis unicolor* USNM 523681, USNM 523682*.

*Loxocemus bicolor* BMNH 82.8.7.16, USNM 348509*.

Pythonidae: *Liasis amethystinus* ROMV R-7507; *Liasis mackloti* ROMV R-7705; *Leiopython albertisii* ROMV R-4076, USNM 523581; *Morelia spilota* BMNH 88.10.27, ROMV R-7508, UCMP 138691, USNM 065471, USNM 523583*; *Python curtus* ROMV R-7289, USNM 94430; *Python molurus* UCMP 123086, USNM 292077; *Python regius* ROMV R-7503; USNM 523587*; *Python reticulatus* UCMVZ 5440, USNM 348606; *Python sebae* BMNH unnumbered, ROMV R-0052, ROMV R-7504.

Boinae: *Acrantophis dumerili* ROMV R-7720; ROMV R-7833, USNM 497683*; *Boa constrictor* ROMV R- 2792, UCMP 126294, USNM 220299*, USNM 348567, USNM 348597; *Candoia carinata* UCMP 187837, UCMP 138260; USNM 348502; *Corallus enydris* UCMP 136964, UCMP 136965; USNM 348502*; *Corallus caninus* ROMV R-398, ROMV R-7498; *Epicrates* *angulifer* ROMV R- 7842; *Epicrates cenchria* ROMV R-5345, ROMV R-6850, UCMVZ 79307, USNM 137839; *Epicrates subflavus* UCMVZ 20000; *Eunectes murinus* BMNH unnumbered, ROMV R- 7285*, UCMP 123085; *Eunectes* *notaeus* ROMV R- 7286*, ROMV R-7307*; *Eunectes* sp. UCMVZ 22986; *Sanzinia madagascarensis* UCMP 137872, USNM 220313*, USNM 166473.

Erycinae: *Calabaria reinhardtii* USNM 523576; *Charina bottae* USNM 009255*, 523755, 523578; *Eryx colubrinus* ROMV R-7292, ROMV R-7293, ROMV R-7519; *Eryx muelleri* USNM 32071; *Lichanura trivirgata* ROMV R-7528, ROMV R-7529.

Tropidophiinae: *Trachyboa boulengeri* BMNH 1901.3.29.6; *Tropidophis canus* USNM 345562*; *Tropidophis haetianus* BMNH 1964-1239; USNM 260730.

Bolyeriidae: *Casarea dussumieri* BMNH 1992.995.

*Acrochordus*: *A. granulatus* USNM 497610*; *A. arafurae* BMNH 1913.10.31.186; *A. javanicus* BMNH 1964.970, BMNH 1964.969.

Colubroidea: *Actractaspis irregularis* USNM 297313*; *Agkistrodon contortix* USNM 330134*; *Bitis gabonica* ROMV R-6845; *Boiga dendrophila* ROMV R-7499; *Boiga irregularis* USNM 331387; *Bothrops nummifer* ROMV R-4078; *Bungarus candidus* ROMV R-100; *Cerberus rynchops* USNM 497588*; *Coluber constrictor* USNM 297253; *Crotalus ruber* USNM 311091*; *Daboia russellii* USNM 297365; *Drymarchon corais* ROMV R-2013; *Erpeton tentaculatum* TMM (VPL) M-8282*; *Farancia abacura* ROMV R-791, ROMV R-2425, ROMV R-2918; *Heterodon platyrhinos* USNM 310959*; *Hydrophis semperi* USNM 499890*; *Lampropeltis getulus* ROMV R-1581, ROMV R-7523; *Lampropeltis* *triangulum* ROMV R-731, ROMV R-6852, ROMV R-7116; *Madagascarophis colubrina* USNM 34553*; *Masticophis flagellum* ROMV R-1646; *Mehelya crossi* USNM 320700*; *Micrurus fulvius* USNM 292571*; *Naja* *nigricollis*, USNM 320722; *Notechis* *scutatus* ROMV R-101; *Oxyrhabdium leporinum* USNM 497051*; *Pituophis melanoleucas* ROMV R-1645; *Ptyas mucosus* USNM 297339;*Thamnophis* *sirtalis*, USNM 326566*; *Waglerophis* *merremii* USNM 523656.

**Literature Cited in TEXT S6**

Scanlon JD (2006) Skull of the large non-macrostomatan snake *Yurlunggur* from the Australian Oligo-Miocene. Nature 439: 839–841.

Apesteguía S, Zaher H (2006) A Cretaceous terrestrial snake with robust hindlimbs and a sacrum. Nature 440: 1037–1040.

Estes R, Frazzetta TH, Williams EE (1970) Studies on the fossil snake *Dinilysia patagonica* Woodward: Part I. Cranial morphology. Bull Mus Comp Zool 140: 25–74.

Frazzetta TH (1970) Studies on the fossil snake *Dinilysia patagonica* Woodward. Part II. Jaw machinery in the earliest snakes. Forma et Functio 3: 205–221.

McDowell SB (1987) Systematics. In: Seigel RA, Collins JT, Novak SS, editors. Snakes: Ecology and evolutionary biology. Toronto: McGraw-Hill Publishing. pp. 1–50.

Caldwell MW, Albino A (2002) Exceptionally preserved skeletons of the Cretaceous snake *Dinilysia patagonica* woodward, 1901. J Vertebr Paleontol 22: 861–866.

Zaher H, Apesteguía S, Scanferla, CA (2009) The anatomy of the Upper Cretaceous snake *Najash rionegrina* Apesteguía & Zaher, 2006, and the evolution of limblessness in snakes. Zool J Linn Soc: doi: 10.1111/j.1096-3642.2009.00511.x

Anthony J, Guibé J (1952) Les affinities anatomiques de *Bolyeria* et de *Casarea* (Boidés). Mém Inst Sci Madagascar, Sér A7: 189–201.

Hecht MK, LaDuke TC (1988) Bolyerine vertebral variation: a problem for paleoherpetology. Acta Zool Cracov 31: 605–614.

Maisano J, Rieppel O (2007) *Casarea dussumieri* (On-line), Digital Morphology. Accessed August 31, 2009 at http://digimorph.org/specimens/Casarea_dussumieri.

Cundall D, Rossman DA (1993) Cephalic anatomy of the rare Indonesian snake *Anomochilus weberi* Zool J Linn Soc 109: 235–273.

Cundall D, Wallach V, Rossman DA (1993) The systematic relationships of the snake genus *Anomochilus*. Zool J Linn Soc 109: 275–299.

Rieppel O, Maisano JA (2007) The skull of the rare Malaysian snake *Anomochilus leonardi* Smith, based on high-resolution X-ray computed tomography. Zool J Linn Soc 149: 671–685.

Rieppel O, Maisano, J (2007) *Anomochilus leonardi*, Accessed August 31, 2009 at http://digimorph.org/specimens/Anomochilus_leonardi.

Kearney M. (2002) Appendicular skeleton in amphisbaenians (Reptilia:Squamata). Copeia 2002: 719–738.

Kearney M. (2003) Systematics of the Amphisbaenia (Lepidosauria: Squamata) based on morphological evidence from Recent and fossil forms. Herpet Monog 17: 1–74.

Gans C, Montero R (2008) An atlas of amphisbaenian skull anatomy. In Gans C, Gaunt AS, Adler K. editors. Biology of the Reptilia, Vol. 21, Morphology I. The skull and appendicular locomotor apparatus of Lepidosauria. New Haven: Society for the Study of Amphibians and Reptiles. pp. 621–738.

The Deep Scaly Project (2006) *Amphisbaena alba* (On-line), Digital Morphology. Accessed August 31, 2009 at http://digimorph.org/specimens/Amphisbaena_alba.

The Deep Scaly Project (2008) *Bipes biporus* (On-line), Digital Morphology. Accessed August 31, 2009 at http://digimorph.org/specimens/Bipes_biporus.

Maisano J (2003) *Loveridgea ionidesii* (On-line), Digital Morphology. Accessed August 31, 2009 at http://digimorph.org/specimens/Loveridgea_ionidesii/.

Gower DJ, Vidal N, Spinks JN, McCarthy CJ (2005) The phylogenetic position of Anomochilidae (Reptilia: Serpentes): first evidence from DNA sequences. J Zool Syst Evol Res 43: 315–320.

Scanlon JD (2005) Cranial morphology of the Plio-Pleistocene giant madtsoiid snake *Wonambi naracoortensis*. Acta Palaeontol Polonica 50: 139–180.

Rieppel O, Kluge AG, Zaher H (2002) Testing the phylogenetic relationships of the Pleistocene snake *Wonambi naracoortensis* Smith. J Vertebr Paleontol 23: 812–829.

Conrad JL (2008) Phylogeny and systematics of Squamata (Reptilia) based on morphology. Bull AMNH: 310: 1–182.

Vidal N, Hedges SB (2004) Molecular evidence for a terrestrial origin of snakes. Proc Roy Soc London B Supp: S226–S229.

Swofford DL (2003) PAUP*. Phylogenetic Analysis Using Parsimony (*and Other Methods). 4 ed. Sunderland, Massachusetts: Sinauer Associates.

Maddison WP, Maddison DR (1992) MacClade, Version 3.0. Sunderland, Massachusetts: Sinauer Associates.

Tchernov E, Rieppel O, Zaher H, Polcyn MJ, Jacobs LL (2000) A fossil snake with limbs. Science 287: 2010–2012.

Frazzetta TH (1966) Studies on the morphology and function of the skull in the Boidae (Serpentes). J Morphol 118: 217–296.

Rieppel O, Head JJ (2004) New specimens of the fossil snake genus *Eupodophis* Rage & Escullié, from the Cenomanian (Late Cretaceous) of Lebanon. Mem. Soc. It. Sci. Natl. Mus. Civ. Stor. Nat. Milano 32: 1–26.

Norell MA, Gao KQ (1997) Braincase and phylogenetic relationships of *Estesia* *mongoliensis* from the Late Cretaceous of the Gobi Desert and the recognition of a new clade of lizards. Am Mus Novitates 3211: 1–25.

Frazzetta TH (1999) Adaptations and significance of the cranial feeding apparatus of the sunbeam snake (*Xenopeltis unicolor*). J Morphol 239: 27–43.

Lee MSY, Scanlon JD (2002) Snake phylogeny based on osteology, soft anatomy and ecology. Biol Rev: 333-401.

Kluge AG (1991) Boine snake phylogeny and research cycles. Misc Pub Mus Zool Univ Michigan 178: 1–58.

Rieppel O (1988) A review of the origin of snakes. Ev Biol 22: 37–130.

Rieppel O (1977) Studies on the skull of the Henophidia (Reptilia: Serpentes). J Zool 181: 145–173.

Kearney M, Rieppel O (2006) An investigation in the occurrence of plicidentine in the teeth of squamate reptiles. Copeia 2006: 337–350.

Alexander AA, Gans C (1966) The pattern of dermal-vertebral correlation in snakes and amphisbaenians. Zool Meded 41: 171–190.

LaDuke TC (1991) The fossil snakes of Pit 91, Rancho La Brea, California. Nat Hist Mus Los Angeles County Contr Science 424: 1–28.
